# Time Series Forecasting of Univariate Agrometeorological Data: A Comparative Performance Evaluation via One-Step and Multi-Step Ahead Forecasting Strategies

**DOI:** 10.3390/s21072430

**Published:** 2021-04-01

**Authors:** Saurabh Suradhaniwar, Soumyashree Kar, Surya S. Durbha, Adinarayana Jagarlapudi

**Affiliations:** Centre of Studies in Resources Engineering, Indian Institute of Technology Bombay, Mumbai 400076, India; saurabh.suradh@gmail.com (S.S.); ksoumya2301@gmail.com (S.K.); sdurbha@iitb.ac.in (S.S.D.)

**Keywords:** precision agriculture, time series forecasting, multi-step ahead forecasting, internet-of-things (IoT), seasonal auto-regressive models, support vector machines, multilayer perceptron, recurrent neural networks, long-short-term-memory, walk-forward validation, temporal bifurcation

## Abstract

High-frequency monitoring of agrometeorological parameters is quintessential in the domain of Precision Agriculture (PA), where timeliness of collected observations and the ability to generate ahead-of-time predictions can substantially impact the crop yield. In this context, state-of-the-art internet-of-things (IoT)-based sensing platforms are often employed to generate, pre-process and assimilate real-time data from heterogeneous sensors and streaming data sources. Simultaneously, Time-Series Forecasting Algorithms (TSFAs) are responsible for generating reliable forecasts with a pre-defined forecast horizon and confidence. These TSFAs often rely on modelling the correlation between endogenous variables, the impact of exogenous variables on latent form and structural properties of data such as autocorrelation, periodicity, trend, pattern, and causality to approximate the model parameters. Traditionally, TSFAs such as the Holt–Winters (HW) and Autoregressive family of models (ARIMA) apply a linear and parametric approach towards model approximation, whilst models like Support Vector Regression (SVRs) and Neural Networks (NNs) adhere to a non-linear, non-parametric approach for modelling the historical data. Recently, Deep-Learning-based TSFAs such as Recurrent Neural Networks (RNNs), and Long-Short-Term-Memory (LSTMS) have gained popularity due to their capability to model long sequences of highly non-linear and stochastic data effectively. However, the evolution of TSFAs for predicting agrometeorological parameters pivots around one-step-ahead forecasting, which often overestimates the performance metrics defined for validating forecast capabilities of potential TSFAs. Hence, this paper attempts to evaluate and compare the performance of different machine learning (ML) and deep learning (DL) based TSFAs under one-step and multi-step-ahead forecast scenarios, thereby estimating the generalization capabilities of TSFA models over unseen data. The data used in this study are collected from an Automatic Weather Station (AWS), sampled at an interval of 15 min, and range over one month. Temperature (T) and Humidity (H) observations from the AWS are further converted into univariate, supervised time-series diurnal data profiles. Finally, walk-forward validation is used to evaluate recursive one-step-ahead forecasts until the desired prediction horizon is achieved. The results show that the Seasonal Auto-Regressive Integrated Moving Average (SARIMA) and SVR models outperform their DL-based counterparts in one-step and multi-step ahead settings with a fixed forecast horizon. This work aims to present a baseline comparison between different TSFAs to assist the process of model selection and facilitate rapid ahead-of-time forecasting for end-user applications.

## 1. Introduction

The ability to generate reliable and ahead-of-time forecasts for agrometeorological parameters is an essential aspect of any agricultural system. In Precision Agriculture (PA), the possibility to extrapolate to future scenarios and approximate the variations in the biophysical responses of crops due to these environmental fluctuations is of the utmost importance. Ahead-of-time forecasts are also crucial for risk minimization in agricultural systems, which rely on empirical data and process-based models to mimic the uncertainty associated with unseen data [1]. Due to this, the relative performance of any Time Series Forecasting Algorithm (TSFA) depends on the methodological robustness of algorithmic assumptions (such as gaussian vs. non-gaussian, structural vs. empirical risk minimization, etc.), the stochasticity of historical data (random vs. stochastic vs. causal) and, finally, the asymptotic complexity while simultaneously modelling complex plant–atmosphere interactions [2,3,4,5].

To simplify the process of modelling and ease of inference, the literature often disentangles the task of forecasting complex phenomenon into smaller constituent forecast sub-problems. For example, physical processes such as Evapotranspiration (ET) can be complex and unreliable to model directly as a short-term process (diurnal) due to high noise and variability within a day [6]. Further, the sub-problem of forecasting constituent elements of ET, such as Temperature, Humidity, Solar Radiation, etc. [7,8,9] can help in recursively modelling ET over a shorter duration. Additionally, the diurnal periodicity and seasonality associated with these elements further simplify the task of multivariate forecasting and can be extended for medium-term forecasts. Considering the task of collecting these variables, the recent upsurge in Internet-of-Things (IoT)-based platforms can be upscaled to design near real-time monitoring systems for seamless sensing and integration of data [10,11,12,13].

One of the major concerns while applying TSFAs on time-series agrometeorological data is the lack of generalization capabilities over unseen data and high reliance on short-horizon forecasts [14,15]. As a generic norm, TSFAs (statistical or machine learning-based) try and optimize a loss function by minimizing the difference between actual and predicted observations by applying suitable linear or non-linear data transformations. Furthermore, the data is projected onto a high-dimensional feature space, wherein the feature space is the set of all observations, also termed as *history* of observations. However, the actual predictive capability of any TSFA is based on two main factors: (a) performance of accuracy measures used for validation over unseen data and (b) ability to generate forecasts over a given forecast horizon.

In the first case, accuracy measures such as Root Mean Square Error (RMSE), Mean Absolute Error (MAE) and Mean Absolute Percentage Error (MAPE) are some of the commonly applied methods in the domain of time-series forecasting. As per standard nomenclature, *training data* are to fit the model parameters, while the *test data* are used to evaluate the forecasting performance of TSFAs. However, an underlying assumption of this *train–test* split, is that the test data (a small fraction of original time series) are a “representative” sample of the total observed dataset [16]. It typically means that statistical assumptions about dataset characteristics, such as gaussian with expected mean (E) and variance (V), remain constant (also termed as stationarity) throughout the training phase and similar rules are valid during model evaluation [17]. Thus, the TSFAs assure that the correlation *learned* during training is also valid during out-of-sample model evaluation [5,18]. Inadvertently, such assumptions are often a source of error in highly stochastic processes such as Relative Humidity.

As an example, a Humidity timeseries can average around 30–40% for a certain period and can saturate to 100% during rainfall, thereby introducing an abrupt change in timeseries that was not *seen* in data before. Due to such short sporadic changes in variance of data (also called heteroskedasticity in the context of linear models), accuracy measures often under or overestimate the performance of TSFAs by including such measurements into modelling process and thereby violating the assumptions of train–test split. These changes further influence model evaluation by incorporating high variances during forecasting, thereby amounting to large errors in forecasts [19]. Nevertheless, these errors are prominent in short-term forecasts and are often averaged out (or smoothened) during long-term (monthly or yearly) forecasting by generating more diversified training data. Hence, a robust yet flexible approach is required to model the forecast error over a fixed forecast horizon to address these issues. Additionally, in this work, the forecasts are referred to as short, medium and long term, based on *the time difference between consecutive measurements*, and not the length of data. This is because two timeseries datasets can have the same length, and yet can represent different temporal frequencies. For example, a series with 96 observations can either represent one-day data with sampling frequency of 15 min or a four days of data with a 1-h sampling interval.

However, in the second case, the length of forecast horizon over which a TSFA can perform reliably is indicative of its forecast performance over unseen data [20,21]. Evidently, the literature around TSFAs for agrometeorological data is constricted around one-step head forecasts (forecast horizon = 1), where previous *n* observations are used to forecast the *n + 1st* value in the series. In the next forecast iteration, the newly predicted value is treated as part of history (training data), and previous *n + 1* observations are used to predict *n + 2nd* value, etc. During this, the accuracy measures perform exceptionally well as, at any given time, TSFAs forecast only *one-step ahead* in future. In contrast, multi-step ahead (forecast horizon = *n*) methods recursively compute one-step ahead forecasts, until the desired forecast horizon is achieved. Due to this, prediction errors at each iteration are accumulated over the length of the horizon, resulting in performance deterioration over time. This study employs walk-forward validation for model evaluation to address this problem, which in turn can make models more reliable over short-term forecasts [22]. In principle, walk-forward validation is similar to k-fold cross validation (Kf-CV), which is frequently used for validating regression models [23]. Walk-forward validation (WFVal) is based on the sliding window method, where the data are consumed strictly in ascending order of time, rather than randomly shuffling train–test datasets. This modelling approach is essential for time-series analysis methods where observations with future timestamp information cannot be used to predict the past (old) values (timeseries data are autocorrelated in time, while regression assumes independence between consecutive observations). Thus, it is crucial to assess model performance by recursively augmenting the training data with recent observations and re-evaluating the model over extended horizon.

Advancements in deep-learning-based forecasting models that focus on ensemble-based approaches for generating reliable forecasts over extended horizon and hence have dominated the recent literature. These models can be broadly classified into four major verticals: a. Feed-forward Neural Network models b. Recurrent models c. Attention-based models or Transformers and d. Graph-based models. In this context, Lim et al. [24] present a comprehensive survey of deep learning models for time series forecasting. The work focuses on models used for sequence-to-sequence time series forecasting by using Convolutional Neural Networks (CNNs), Recurrent Neural Networks (RNNs) and Attention-based models with a brief overview of direct and iterative strategies for forecasting multiple steps in future. Dabrowski et al. [25] proposed a time-invariant deep feed-forward neural network architecture which was evaluated over synthetic dataset and nine real-world datasets for multi-step ahead forecasting. The authors further compared the proposed model’s performance against autoregressive, sequence-to-sequene and attention-based models [26]. Hewage et al. [27] demonstrated the utility of deep-learning-based models for generating fine-grained weather data and compared the results against weather research and forecasting model (WRF) [28]. The authors explored different architectural variations of long short-term memory (LSTMs) and Temporal Convolutional Networks (TCN) with multi-input, multi-output (MIMO) and multi-input, single-output (MISO) configurations for time series data. Ferreira et al. [29] compared the performance of long short-term memory (LSTMs), one-dimensional convolutional neural networks (1D-CNN) and an ensemble-based approach using CNN-LSTM architecture for estimating daily reference evapotranspiration (ET_0_). In addition, the study also presents the performance of forecasting models using iterated or recursive, direct and multi-output forecast strategies for multi-step ahead forecasts. Hu et al. [30], proposed a multi-stage attention network with influential and temporal attention to understand the influence of non-predictive variables on forecasting of multivariate time series data. For this, the authors proposed a transformation-gated LSTM (TG-LSTM) and compared the model performance against nine different recurrent and attention-based models. In the recent times, Graph Neural Networks (GNNs) have gained popularity in multivariate time series forecasting where temporal and latent interdependencies between variables can be represented using graphs with nodes as variables and edges representing the dependencies between variables [31,32]. Unarguably generating ahead-of-time forecasts for financial time series is one of the most challenging and widely studied problems in the domain of time series forecasting. Moody et al. [33] present some of the early work that uses reinforcement learning for trading applications. Recent trends include work from Carta et al. [34], where the authors propose an ensembled-based approach to using reinforcement learning for stock market predictions. Similarly, Luo et al. [35] proposed a hybrid model for financial time series forecasting using empirical model decomposition, autoregressive family of models (ARIMA) and Taylor expansion. Li et al. [36] showcased that an ensemble of ARIMA–LSTM can improve the forecasting accuracy in high-frequency financial time series. Going forward, Cappelli et al. [37] emphasize the importance and detection of multiple breakpoints in financial time series, while Niu et al. [38] proposed a two-stage deep learning framework focusing on feature selection for multivariate financial time series.

Several studies have addressed the issue of generating reliable one-step and multi-step ahead forecasts for weather parameters using a multivariate approach for modelling and generating forecasts. However, this work stands-out from the existing literature and focuses on univariate time series analysis due to two main reasons: a. Univariate forecast models are simple to train with limited data and provide ease of inference while evaluating the forecast performance. As the physical processes associated with agrometeorological data have complex interactions, it is simpler and efficient to forecast the variables individually. In contrast, more complex processes can be approximated empirically using physics-based models that are well-studied, are more robust and generalise exceptionally well under different global conditions. b. The models used in this work are envisaged to consume real-time data from sensors and generate on-field inference (forecasts) on Edge devices. As different user applications can necessitate additional sensory data requirements, a singular multivariate modelling approach that is designed using a fixed set of parameters may not suffice. On the contrary, time series data streaming from different sensors can be consumed by individual univariate models to generate forecasts on edge devices in real-time. Simultaneously, a more complex phenomenon can be approximated off-line on remote or cloud resources.

Nevertheless, the choice of one-step vs. multi-step forecasts is often dependent on the end-application and is always an algorithmic consideration. For example, one-step ahead forecasts might be a right choice for long-term (like monthly or daily average) estimation of ET, where small variations within the data are averaged out due to their negligible influence on atmospheric exchange. However, to understand the transpiration rate of plants under stress (also associated with ET, but at shorter interval), multi-step ahead forecasts might be a better fit for modelling diurnal variations [39,40,41]. Finally, exogenous factors such as dynamics of observed phenomenon and interaction between involved processes directly impact the complexity of associated methods and thus the choice of TSFAs.

To address the above issues persistent with the application of TSFAs, this work makes the following novel contributions over the existing literature:

1. Quantitative assessment of *n-step* ahead forecasting capabilities of statistical and machine-learning-based Time Series Forecasting Algorithms (TSFAs) using univariate agrometeorological datasets.
2. Application of recursive approximation, walk forward validation and fixed forecast horizon in evaluating model performance.
3. Validate the forecast proficiency of TSFAs over fixed temporal partitioning of the dataset while evaluating the average performance of models over entire dataset.

The rest of the paper is organized into the following sections: Section 2 describes the methodology of work, dataset description and a formal introduction for TSFAs used in this study. Section 3 puts forward the significant results and findings of this work and in-depth discussion on various aspects of using forecasting models on an agrometeorological dataset. Finally, Section 4 presents the conclusion and potential for extending the current scope of work.

## 2. Materials and Methods

This section provides an in-depth description of the materials and methods used throughout this study. The first subsection briefly describes the test site and dataset characteristics. Subsequent subsections present the conceptual background for recursive estimation of one-step-ahead vs. multi-step-ahead forecasts, followed by a formal introduction to TSFAs. Finally, it explains the intuition behind using these methods for time series forecasting.

### 2.1. Test Site and Dataset Description

This study’s dataset is collected from an automatic weather station (AWS) installed at Bargaon village (21.4427° N, 78.1335° E) in the Vidarbha region of central India. As per Köppen–Geiger classification [42], the area falls under the tropical savannah climate zone, characterized by long dry-spells in summers, followed by short but extreme flash rainfall during the south-western monsoon season (Jul-Sep). In addition to this, the region falls into a typical semi-arid climatic zone with mean annual precipitation of about 800–900 mm. Average daily maximum temperatures in the area range from 32 to 42 °C, occasionally rising to 48 °C, during summers, while average daily minimum temperature ranges from 15 to 24 °C during winters. The selected study area is also one of the largest Citrus growing regions of central India [43].

A total of 6 parameters, Temperature (T), Humidity (H), Rainfall (R), Solar Radiation (Rad), Wind Speed and Direction (WS), Atmospheric Pressure (P) along with timestamp information were obtained from the AWS in comma separated values (CSV) format. Each of these parameters was sampled at 15 min interval and accumulated over 60 min (four observations per hour), for further transmission to the remote server. For this study, the Temperature and Humidity observations from 01 February to 29 February (a total of 29 days) were separated and converted into univariate time-series profiles. After pre-processing and initial data cleaning, a total of 2784 data points (96 datapoints/per day @ 15 min interval for 29 days) were obtained for modelling. The month of February was explicitly chosen for the study, as it showcased high heteroskedasticity and sporadic structural changes in weather, associated with the seasonal transition from winters to summers, with intermittent rainfall events. Before application of TSFAs, data were pre-processed to remove any outliers or missing values /timestamp information. After this, algorithm-specific processing such as data transformation, conversion to supervised time series, dimension matching and batch generation were applied before final model generation.

### 2.2. Background

#### 2.2.1. One-Step vs. Multi-Step Ahead Forecasting

The task of timeseries forecasting, where a dependent variable $y_{t}$ can be represented as a function of auto-correlated features $x_{t-1}$, regressed over the parameter space θ and where the residuals $\epsilon_{t}$ are independent and identically distributed, is shown below:

(1) $$y_{t}=f\left(x_{t-1};\theta \right)+\;\epsilon_{t}\;$$

A modified version of Equation (1), given as a univariate time series $\left\{y_{1},\;y_{2}\ldots y_{t}\right\}$, comprising of t observations and h be the forecast horizon, then assuming the data comes from a possibly non-linear autoregressive process of form [44]:

(2) $$y_{t}=f\left(x_{t-1}\right)+\;\epsilon_{t}\;\mathrm{with}\;x_{t}={\left[y_{t},\;\ldots \;y_{t-d+1}\right]}^{\prime }$$

where the forecast generating process is specified by the function f, embedding dimension d, and error term $\epsilon_{t}$, the conditional mean can be represented as:

(3) $$\mu_{t+h|t}=E\left(y_{t+h}|x_{t}\right)$$

The *recursive* strategy [45,46] minimizes the squares of the in-sample one-step ahead residuals and uses the newly predicted value as an input to the same model f to forecast subsequent data points until the desired prediction horizon is achieved. Thus, using Equations (2) and (3), the values for Mean Squared Error (MSE) at forecast horizon h can be approximated as an additive model with noise, bias and variance [44,47,48]:

(4) $$MSE_{h}=\;E[{\left(y_{t+h}-{\hat{f}}^{\left(h\right)}\left(x_{t}\right)\right)}^{2}]\;\begin{array}{c} \;=\;E[{\left(y_{t+h}-\;\mu_{t+h|t}\right)}^{2}]+{\left(\mu_{t+h|t}-\;f^{\left(h\right)}\left(x_{t}\right)\right)}^{2}+E[{\left({\hat{f}}^{\left(h\right)}\left(x_{t}\right)-\;f^{\left(h\right)}\left(x_{t}\right)\right)}^{2}] \end{array}$$

where ${\hat{f}}^{\left(h\right)}\left(x_{t}\right)$ and $f^{\left(h\right)}\left(x_{t}\right)$ denote the forecast generated by function f over forecast horizon h and actual observations, given the series $\left(x_{t}\right)$. After learning the minimization parameters and accounting for bias and variance of expected and predicted observations, the forecasts pertaining to different horizons can be written as:

(5) $${\hat{y}}_{t+h}=\;\left\{\begin{array}{l} \hat{f}(y_{t},\;\ldots \;y_{t-d+1)}\;\mathrm{if}\;h=1\\ \hat{f}\left({\hat{y}}_{t+h-1},\;\ldots \;{\hat{y}}_{t+1},\;y_{t},\;\ldots \;y_{t-d+h}\right)\;\mathrm{if}\;h\in \left\{2,\;\ldots d\right\}\\ \hat{f}\left({\hat{y}}_{t+h-1},\;\ldots \;{\hat{y}}_{t-d+h}\right)\;\mathrm{if}\;h\in \left\{d+1,\;\ldots h\right\} \end{array}\right.$$

where $y_{t}$ and ${\hat{y}}_{t}$ denote actual and forecasted values at time t, forecast horizon is h and embedding dimension is d. The case where, h=1, is classically termed as “one-step ahead” forecast, whilst the other two cases denote “multi-step ahead forecasts”, which are calculated by recursively approximating one-step ahead forecasts and augmenting training data with recently forecasted values over forecast horizon [49]. The embedding dimension d, is latent representation of actual data that can either comprise of prominent features identified from the dataset or batch-size of data used during each iteration. The objective of using such notation is to simplify model formulation and easy of model representation.

**Figure 1:**
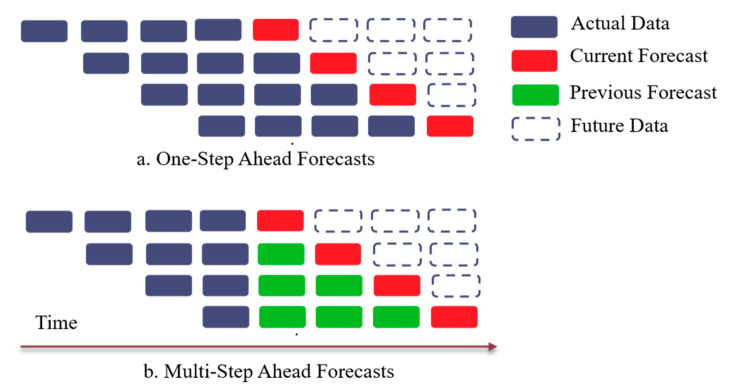
(**a**) One-step ahead forecasting where at each step *forecast horizon = 1* and *window size = w* (**b**) Multi-step Ahead Forecasting where at each step *forecast horizon = h*, window size = w and the window of predictor variables is saturated with forecasted values after *w* iterations.

**Figure 2:**
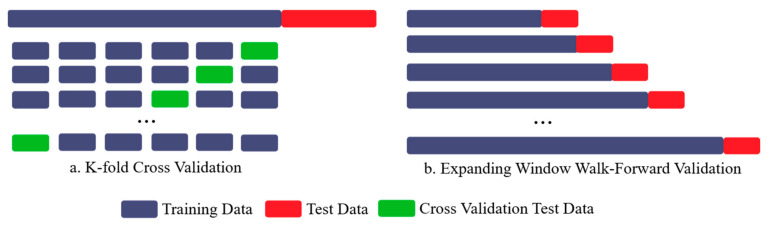
(**a**) Standard K-fold cross validation (Kf-CV) model with training and test data folded into randomly shuffled subsets and hold-out test data for model evaluation (**b**) Walk-forward Validation (WFVal) scheme with adaptive training windows that expand after each iteration to include recent observations.

Figure 1 depicts the difference between one-step and recursive multi-step ahead forecasts adopted in this study. It is apparent that recursive multi-step forecasts suffer from low performance when the forecast horizon h is greater than the dimensional embedding d. This is because, for the long forecast horizon, all the values in the predictor variables in the functional form of timeseries (Equation (5)) are pure “forecasts”. Thus, after each iteration, residuals from the minimization of MSE are accumulated into the original series (in case of additive models), thereby rapidly deteriorating the accuracy estimates. To avoid this, walk-forward validation splits the timeseries into pre-defined sub-segments (usually based on sampling frequency), and the recursive forecast is applied over expanding timeseries segments, generated using previous subset (history) and most recent observations (Figure 2).

#### 2.2.2. Walk Forward Validation and Data Bifurcation

Conventionally, the entire data are split into train–test datasets, where training data are used to estimate the model parameters. In contrast, test data are used to evaluate model performance using cross-validation. To ensure the robustness of the evaluation criterion, the process of validation is repeated several times, often called K-fold cross-validation (Kf-CV). However, while using Kf-CV it is assumed that the observations are mutually independent. Contrary to this, timeseries datasets are correlated in time and often have temporal interdependencies. Due to this, one-step ahead forecasts tend to become biased over time as each forecast can be approximated using previous forecast plus some error. Due to this, the model presents unrealistically optimistic values for evaluation matrix over the one-step horizon. As for multi-step ahead forecasts generated recursively, the predictions quickly become erroneous with an increase in the prediction horizon. As discussed before, these issues are partially solved using a walk-forward validation (WFVal) approach with expanding window methodology, where the size of the forward window is based on the sampling frequency of time series or embedded dimension d. By using a window-based approach, training and validation sets are iteratively time-shifted to incorporate recent observations, thereby refreshing the forecast window (based on the horizon) after each iteration. The differences between standard K-fold cross-validation and walk-forward validation are presented in Figure 2.

The key advantages of using the walk-forward method over k-fold cross-validation are two-fold. Firstly, WFVal does not assume that *entire* dataset is readily available for processing, thereby offering flexibility of iteratively augmenting the training data (history) with more recent observations before making predictions. Kf-CV employs a similar approach, but with added computations for k-fold cross validation that does not adhere to auto-correlation in observations. Secondly, in WFVal the selection of *split-point* defines the bifurcation of train–test data based on the sampling frequency, and eventually on the model update frequency of the underlying sensing platform. In simple terms, the split-point of walk-forward validation depends on how frequently the forecasting model parameters are updated. Whereas in the case of Kf-CV, the split-point is often a fixed ratio partitioning (70–30), creating non-representative test sets, thereby generating sub-optimal forecasts [16]. In the preview of modern IoT-based sensing platforms where data are collected in real-time from streaming data sources, a simplified approach that periodically updates model parameters, while maintaining temporal significance of data, seems more intuitive [50].

In this study, the WFVal approach first uses 192 observations (sampled at 15 min interval over a period of 48 h) to restructure the observations into a supervised learning problem. Here, the timeseries features are self-lagged values of timeseries data, where the number of features is decided based on the forecast horizon. From Equation (5), it is evident that a forecast horizon of 96-steps requires at least 192 observations to generate a labelled dataset with 96 features and one predicted variable, while sliding the forecast window one observation per forecast iteration. After each iteration, the training window for one-step ahead is shifted one time-step by augmenting the actual observation, and the model is re-trained. On the contrary, for multi-step ahead forecasts, the training window is augmented with the recently generated forecast. Each forecast iteration (expanding window) lasts until all the training window observations are replaced by forecasts. Through this approach the underlying TSFA is updated once in every 24 h (horizon of 96), thereby maintaining the model robustness and accuracy over time. These explicit parameters can be modified to match the sensing platform’s sampling interval, thus simplifying the adaptive re-training of the model.

### 2.3. Time Series Forecasting Algorithms (TSFAs) Modelling

A simple case of TSFA is a regression model that assumes a linear relationship between the predictor and predicted variables. As this work considers univariate analysis, a regression model can be defined as:

(6) $$y_{t}=\;\alpha_{0}+\;\alpha_{1}x_{t-1}+\;\varepsilon_{t}\;$$

where $\alpha_{0}\;\mathrm{and}\;\alpha_{1}\;$ are the intercept and slope of the regression fit and $\varepsilon_{t}$ represents the error or “residuals” that are non-autocorrelated and are independent of predictor variables. The values of regression coefficients, $\alpha_{0}\;\mathrm{and}\;\alpha_{1}$, can be estimated using the training data and minimizing the sum of squared errors over predicted values. Finally, a goodness-of-fit measure such as $R^{2}$ is used to assess the forecast accuracy over test/out-of-sample data. This simplistic approach of model estimation and evaluation is further generalized for different TSFAs. Before comparing the performance of different TSFAs, the subsequent section presents a brief mathematical foundation for TSFAs used in this study.

#### 2.3.1. Seasonal Auto-Regressive Integrated Moving Average (SARIMA)

Seasonal ARIMA models are linear, parametric models that combine seasonal and non-seasonal components of data and model the variations using auto-regressive and moving-average terms. A typical ARIMA (non-seasonal) process can be defined as:

(7) $$ARIMA\;\left(p,\;d,\;q\right)\;y_{t}=c+\emptyset_{1}y_{t-1}+\ldots \;\emptyset_{p}y_{t-p}+\theta_{1}\varepsilon_{t-1}+\ldots \;\theta_{q}y_{t-q}+\;\varepsilon_{t}\;$$

where p and q represent the AR and MA terms, d defines the level of differencing and the coefficients ∅ and θ are estimated using training data. Similarly, a Seasonal ARIMA model can be represented as $ARIMA\;\left(p,\;d,\;q\right){\left(P,D,Q\right)}_{m}$, where m represents the seasonal period and P, D and Q are seasonal variants of AR and MA terms with differencing D [51]. Before modelling the data using SARIMA, the time series is checked for stationarity using the Kwiatkowski–Phillips–Schmidt–Shin (KPSS) test statistic where the null hypothesis assumes that data are stationary in nature. Post this, auto-correlation (ACF) and partial auto-correlation (PACF) plots are used to identify initial AR and MA model parameters. The model coefficients are estimated recursively using maximum-likelihood estimation (MLE), where the number of model parameters are regularized using Akaike’s information criterion (AIC). Finally, the Ljung-Box test, where null hypothesis assumes that residuals are non-correlated, is applied to ensure that residuals resemble white noise and does not include any significant auto-correlations in previous lags. The SARIMA model is implemented using *pmdarima* and *statsmodel* libraries in the Python and *forecast* package from R studio [52].

#### 2.3.2. Support Vector Regression (SVR)

Support Vector Regression is widely used in time-series forecasting due to its ability to model non-linear interaction between predictor variables using kernel methods to construct non-linear decision boundaries [53]. The standard Epsilon-SVR (ϵ-SVR) uses an epsilon-insensitive hinge loss function to define the margin of tolerance for penalizing the prediction error. The objective function of SVR can be defined as:

(8) minw, b, ξ, ξ*12 w2+C∑i=1m(ξ+ξi*) s.t.yi−wTxi+b≥ ϵ+ξi i=1, …,m (wTxi+b)− yi ≥ ϵ+ ξi*i=1, …m ξ,  ξi* ≥0, i=1, …,m 

where slack variables $\xi ,\;\;\xi_{i}{}^{*}$ provide a level of tolerance for observations lying outside the ϵ-insensitive tube, however, penalizes any observations out of this region. The above optimization problem is often solved using a dual-form representation, while using a kernel function to project the data into a higher-dimensional vector space. This study uses a Radial Basis Function (RBF), where $K\left(x_{i},\;x_{s}\right)=\exp(\gamma -\;{\left|\left|\;x_{i}-\;x_{s}\right|\right|}^{2}$) and is known to perform better than linear kernel on non-linear datasets. As part of pre-processing, the dataset was restructured into a supervised learning problem by augmenting the dataset with temporally lagged variables. The SVR function was implemented using *sklearn library* in Python.

#### 2.3.3. Multilayer Perceptron (MLP)

Multilayer Perceptron is a non-linear, non-parametric class of models that are often called universal approximators, due to their capability to model highly stochastic processes and no prior assumptions on data statistics or distribution [54]. An MLP model typically consists of an input layer representing input features, a hidden layer that applies a non-linear activation on input features and an output layer with a linear or probabilistic activation function and can be represented as:

(9) $$z_{l,i}=\;\sigma \;\left(w_{l,i}^{T}\;x_{l-1}+b_{l,\;i}\right)\;$$

where $z_{l,i}$ denotes the output of layer l of MLP, $w_{l,i}^{T}$ is the weight matrix for current layer, $x_{l-1}$ is the input from the previous layer, $b_{l,\;i}$ is the bias of the current layer and σ is an activation function. In each forward pass, MLP uses a squared error loss function and a L2 norm for regularizing the weights of the hidden layer. After each epoch, the weights of the hidden layer are updated using the loss function and parameters of the gradient decent algorithm used for backpropagation. In this work structure, MLPs are designed using following guidelines: (a) input features are a multiple of 96, thereby windowing the data into multiple batches. (b) with N as the number of input features, the number of hidden units are assumed to be 2N + 1. The weights are initialized using Xavier initialization and an ADAM optimizer is used with default learning rates for gradient decent, as it performs well under non-stationary objectives and noisy or sparse gradients. Finally, rectified linear units (ReLU) are used as activation functions in hidden layers, while total epochs set to 500 iterations. The MLP functionality was implemented using the *sklearn* library in Python.

#### 2.3.4. Simple Recurrent Neural Networks (RNN)

Simple Recurrent Neural Networks, also called Vanilla or Elman networks, are a logical extension of MLPs which can learn temporal dependencies in input data and are often used in sequence prediction tasks [54,55]. In a typical MLP architecture feedforward network generates an output based only on the current input and activation function. Due to this, there is no notion of context or memory in MLPs. In contrast, the output of RNNs depends on the previous hidden state, the current input and the applied activation function.

(10) $$\begin{array}{c} h_{t}=\;\sigma_{h}\;\left(W_{h}\;x_{t}+\;U_{h}\;h_{t-1}+\;b_{h}\right)\\ y_{t}=\;\sigma_{y}\;\left(W_{y}\;h_{t}+\;b_{y}\right) \end{array}$$

where current hidden state $h_{t}$ is a function of current weighted input $x_{t}$, weighted previous hidden state $h_{t-1}$ and a bias for current hidden layer. Additionally, the current output $y_{t}$ is a function of the current state $h_{t}$ and current bias $b_{y}$. This recurrence in RNNs, enables the network to make predictions that no longer require lagged variables and can easily model trends and seasonality in data. Based on the model definition, an RNN can have different cardinalities such as one-to-one, many-to-one, etc. In this study, a simple RNN with many-to-one architecture is used for recursively generating forecasts. Functionally, RNNs can predict an entire sequence as output; however, it also introduces a modelling bias inherent with multi-output schemes, when comparing against models that do not support sequential output (such as ARIMA and SVR). The RNN functionality was implemented using *simpleRNN* blocks available with *sklearn* library in Python with sequential modelling.

#### 2.3.5. Long-Short Term Memory (LSTM)

As RNNs use a feedback loop to maintain the previous state information, the training of the model becomes slower and difficult with increases in the depth of the network. When the RNN model is trained using gradient descent and temporal backpropagation, the partial derivates during backward traversal in the network generate a complex chain of continuous matrix multiplications. Due to this, if the derivates are large, the updated gradient increases exponentially, resulting in model collapse. This is often termed as *exploding gradients*. On the contrary, if the derivates are too small, the gradient can decrease exponentially, resulting in *vanishing gradients*, where the model stops learning even with an increase in training epochs. Long Short-Term Memory (LSTMs) solves this problem by using dedicated gates to retain the information of past hidden states in “memory cells” and regulate the flow of information by using update and forget gates [56]. Through this, LSTMs can retain longer sequences of data and can model local and temporal dependencies while avoiding model collision due to gradient updates. A simple LSTM model can be represented as:

(11) $$\begin{array}{c} \hat{c_{t}}=\tanh\left(W_{c}\;\left[h_{t-1},\;x_{t}\right]+\;b_{c}\right)\\ \Gamma_{u}=\;\sigma \;\left(W_{u}\;\left[h_{t-1},\;x_{t}\right]+\;b_{u}\right)\\ \Gamma_{f}=\;\sigma \;\left(W_{f}\;\left[h_{t-1},\;x_{t}\right]+\;b_{f}\right)\\ \Gamma_{o}=\;\sigma \;\left(W_{o}\;\left[h_{t-1},\;x_{t}\right]+\;b_{o}\right)\\ c_{t}={\;\Gamma }_{u}*\;\hat{c_{t}}+{\;\Gamma }_{f}*\;c_{t-1}\;\\ h_{t}={\;\Gamma }_{o}*\tanh\left(c_{t}\right) \end{array}$$

where $\hat{c_{t}}$ is internal memory state which is a function of previous hidden state $h_{t-1}$ and current input $x_{t}$. $\Gamma_{u},\;\Gamma_{f}\;\mathrm{and}\;\Gamma_{o}$ represent update, forget and output gates, $c_{t}$ is the new internal memory state that combines fraction of previous internal state regulated by update gate and previous hidden state regulated by the forget gate. Finally, the new hidden state $h_{t}$ is a function of the output gate and hyperbolic tangent of current internal memory. The notations $W_{x}\;\mathrm{and}\;b_{x}\;$ denote weights and bias terms for each gate in the LSTM cell. The LSTM functionality was implemented using LSTM blocks available in *sklearn* library in Python.

### 2.4. Accuracy Measures for Model Evaluation

Accuracy measures used for evaluating models’ forecast performance should be model agnostic and should avoid any potential bias during model training and approximation. Thus, this study uses two evaluation metrics: Root Mean Squared Error (RMSE) and Symmetric Mean Absolute Percentage Error (sMAPE), as defined in Equations (12) and (13), respectively. Here, ${\hat{y}}_{t}\;\mathrm{and}\;y_{t}$ donate the forecast and actual observations, while n denotes the total number of observations used.

(12) $$RMSE=\;\sqrt{\frac{1}{n}\;\overset{n}{\underset{t=1}{\sum }}{\left({\hat{y}}_{t}-\;y_{t}\right)}^{2}}\;$$

(13) $$sMAPE=\;\frac{1}{n}\;\overset{n}{\underset{t=1}{\sum }}\frac{\left|{\hat{y}}_{t}-\;y_{t}\right|}{\left(\left|y_{t}\right|+\;\left|{\hat{y}}_{t}\right|\right)/2}\;$$

### 2.5. Intuition for Walk-Forward Validation and Representative Train-Test Split

Performance estimation of TSFAs depends on the accuracy of forecasts over unseen or out-of-sample data points. Additionally, it also depends on the cumulative error over a multi-step prediction horizon. Consequently, the objective of TSFAs is to provide accurate and reliable forecasts over a given period. However, this is trivial when the entire dataset can be defined using an underlying distribution, which can assist parameter estimation by making prior assumptions on the distribution of new datapoints. On the contrary, for most real-time datasets, this assumption does not hold due to the highly stochastic and non-linear nature of agrometeorological parameters and associated interactions. Under such circumstances, a mixture model that can generate probabilistic partitioning of data using subpopulation distributions seems more intuitive [16]. For this, it is assumed that different subsets (partitions) of data may originate from different distributions and require model re-evaluation after each forecast iteration, thus limiting the usability of single partitioning of data [18].

To verify the above assumption, the entire dataset was split into four consecutive data partitions named from P1 to P4, each spanning over a period of 7 days with non-overlapping timestamps. Each partition is labelled as *Temp_X* and *Hud_X* for temperature and humidity timeseries, respectively, where X denotes the partition number. Further, the Kruskal–Wallis *H* Test (KW) [57] was applied over each of the data partitions. The KW test is a non-parametric variant of one-way analysis of variance (ANOVA) method, which is used to test whether different samples in a subspace originate from the same distribution or not. The KW test can be applied over multiple samples of different lengths and thus is much more flexible compared to the Mann–Whitney U test. A significant value of the KW test (*p*-values) denotes that at least one sample in the sample subspace is significantly different from others. However, it does not identify the group which has significant dominance over other samples. For this, Nemenyi post hoc test [58] is applied after the KW to test the largest pairwise difference between group ranks mean. The results for KW and Nemenyi tests are shown in Table 1.

**Figure 3:**
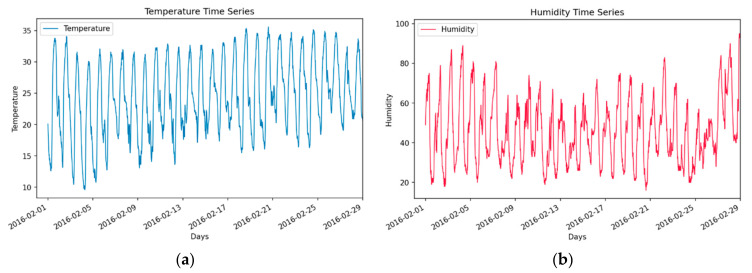
Pre-processed univariate timeseries datasets collected over a period of one-month (1 February 2016 to 29 February 2016) (**a**) Temperature Time Series (**b**) Humidity Time Series.

Temporal partitioning of the dataset can provide useful insights into the data and help design a robust training–test dataset for model training and evaluation. This presumption is verified from Table 1, which shows that subsets Temp_2 and Temp_3 of temperature timeseries are closely related in terms of original data distribution, with a post hoc value of 0.943 (values in **bold**). However, a similar assumption does not hold for partitions Temp_0 and Temp_2, that have a similarity value of 0 (values in *italics*), and thus originate from significantly different distributions. Similarly, for humidity time series, partition Hud_0 is closely related to Hud_2 with a similarity value of 0.654, marginally related to Hud_3 with a value of 0.086 and significantly different from Hud_1, with a value of 0. These results reinforce the opinion that using walk-forward validation is a better alternative when test data may not be a representative sample of the entire dataset and are significantly different from data used for training. The correspondence between the above values and actual timeseries becomes more evident from Figure 3, where observations from the first half of temperature timeseries are pointedly different (mean and variance) from the second half of series. Similarly, in the third quarter of the humidity timeseries a drastic drop in humidity is followed by complete saturation resulting from rainfall in the fourth quarter of the month.

## 3. Results and Discussions

### 3.1. Univariate Timeseries and Seasonal and Trend Decomposition Using Loess (STL) Decomposition

The multivariate dataset obtained from the AWS was pre-processed for any missing or outlier values, followed by extraction of temperature and humidity profiles for further modelling. As mentioned before, this work focuses on estimating the multi-step ahead forecasting capabilities of TSFAs and currently does not consider the impact of regressing against multiple predictor variables. Additionally, as temperature and humidity are known to be influencing variables in the estimation of Evapotranspiration (along with solar radiation), we focus our attention on the approximation of these variables only. Figure 3a,b show the pre-processed univariate timeseries observations for temperature and humidity over one month. Initial assessment of timeseries shows that temperature is more consistent over time and is slightly trending with strong seasonality. It is interesting to notice that towards the end of time series data, a rapid increase in humidity, does not immediately trigger changes in temperature. This is typical for a semi-arid region where temperature has a slow response towards change in humidity due to the high vapor pressure deficit. It also deviates mostly around a mean of 24.52 °C, with a standard deviation of 5.94 °C. In contrast, humidity exhibits large variations and heteroskedastic behavior with a mean of 46.88%Rh and standard deviation of 17.61%.

To further understand the influence of data partitioning and intuition for walk-forwards validation, mean and standard deviations for fixed partitioning of the dataset is shown in Table 2. For humidity, mean for partitions P1 (48.05) and P4 (51.47) tend to deviate positively from the average mean (46.88), whereas partitions P2 (42.17) and P3 (45.78) are below the average mean. Thus, any TSFA that depends on the mean of timeseries for predictive modelling (such as ARIMA or RNNs) can result in damped-out performance due to suppressing mean over average values. For Autoregressive models, this inherently violates the assumption of stationarity over time, while for recurrent models it impacts the backpropagation through time and can result in vanishing gradients. Additionally, the difference between SD around these partitions (P1 = 18.73 P4 = 20.82) and average SD (17.61) suggests that the observations are more dispersed out from the mean, thereby increasing the variance between observations. This inversely impacts the generalization capabilities of TSFA and the models either tend to overfit or have a higher bias. Similar conclusions can be made for temperature timeseries, where the mean of partitions P3 (26.01) and P4 (26.21) trends positively from the average mean. However, these changes are more subtle, resulting in a slightly trending series, and is disassociated with variations in SD. Due to this, in general, the forecasting performance of TSFAs for multi-step ahead temperature forecasting outperforms forecasting results for humidity timeseries (Table 3).

**Figure 4:**
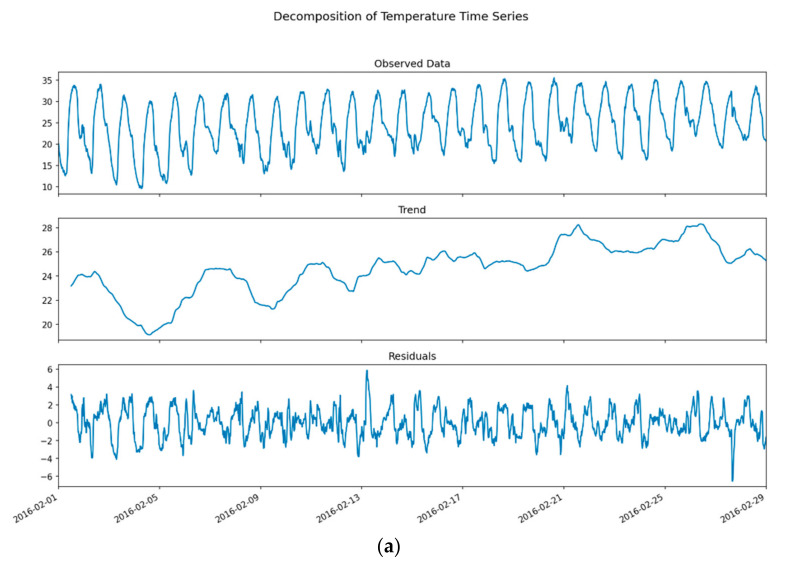

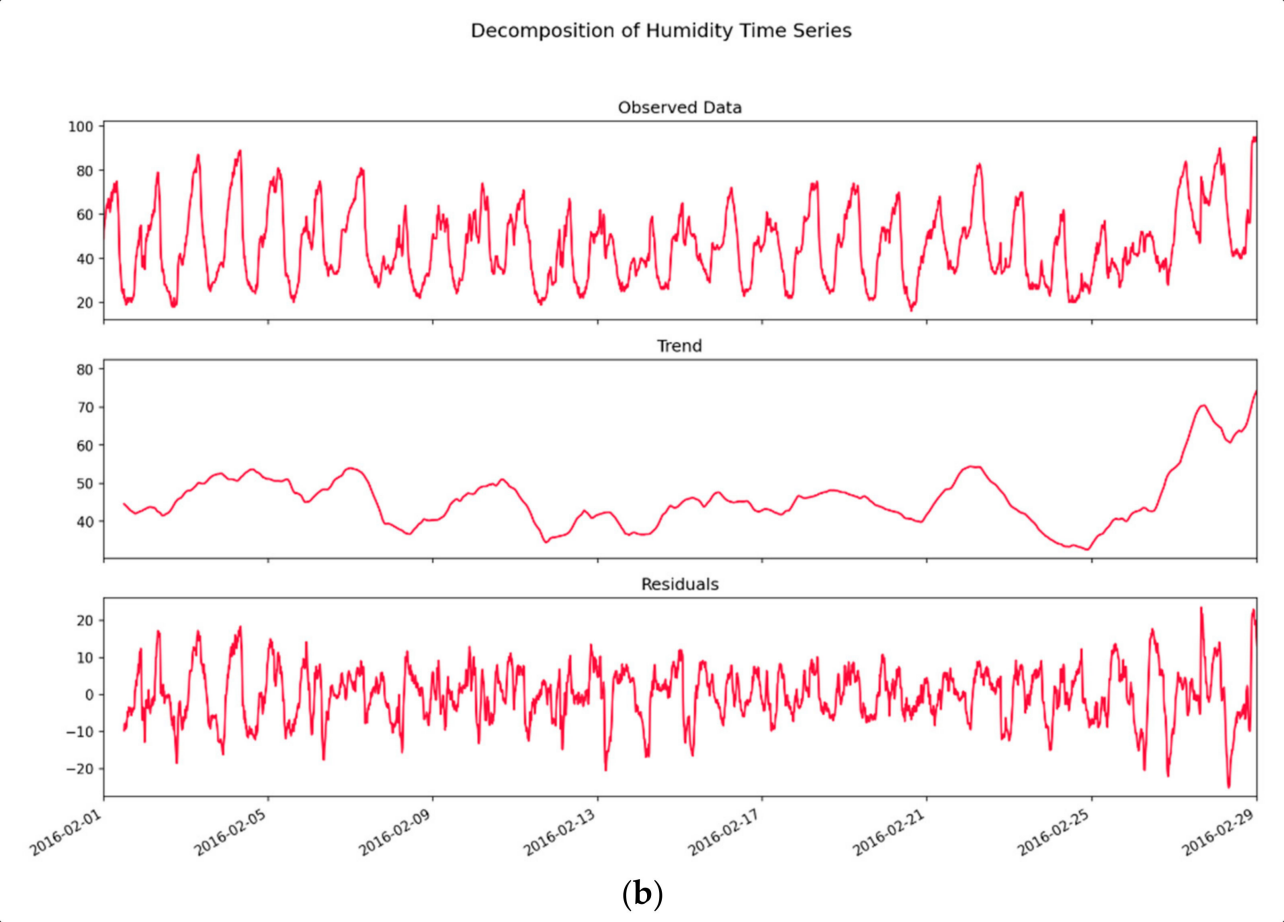
Seasonal and Trend Decomposition with Loess (STL) decomposition of timeseries datasets (**a**) Decomposed Temperature Time Series (**b**) Decomposed Humidity Time Series.

Furthermore, to assess the variational components of seasonality and trend in the dataset, each timeseries is decomposed using STL. The core idea behind decomposition models is to understand the seasonality, trend and residuals of time series. This helps in making appropriate assumptions while training autoregressive models. For example, change in “Trend” (upward or downward) signifies the need for stationary tests and time series differencing. “Residuals” help identify and remove any auto-correlation between model residuals thereby removing any bias in forecasts. It is often useful to model a depersonalized time series, generate forecasts and then add back the seasonal component to achieve final forecasts. Figure 4a shows that the variations in temperature trend upwards while exhibiting diurnal seasonality during the first half (P1 and P2), whereas the variations in the second half of data are more subtle. It also reveals interesting patterns during the above period, where the variations during period P1 portray diurnal seasonality, thereby resulting in highly seasonal and correlated residuals. Similarly, Figure 4b shows that the initial variations in humidity during period P1 are inconsistent with the variations in temperature (a drop in temperature does not influence the relative humidity). This phenomenon is attributed to the dry winters’ characteristic of semi-arid regions. However, the later observations are more coherent, while rapidly deviating towards the end of the experiment window (P4). This rapid change in humidity is attributed to rainfall during the mentioned period. Although, this results in a rapid change in variance and extremely large residuals of decomposition. Due to this stochasticity, TSFAs tend to perform poorly over long forecast horizons in humidity series, where forecasts quickly become unreliable over time.

### 3.2. Forecast Generation Using Time Series Forecasting Algorithms (TSFAs)

Forecasting capabilities of TSFAs largely depend on their ability to model the stochasticity in observed data, the mathematical formulation for forecasting, prior assumptions, and hyperparameter tuning during model training. Due to a multitude of factors, assessing the performance of structurally different models such as SRIMA and RNNs, poses a unique challenge. Thus, model-specific variations and assumptions that mitigate the inter-model variability are crucial. For this, the forecasting performance of different models is compared using the Root Mean Squared Error (RMSE) values to minimize the out-of-sample testing error, while symmetric Mean Absolute Percentage Error (sMAPE) was used to assess the percentage error in predictions. This section presents forecasts generated by TSFAs over one-step, and multi-step ahead (96 steps) forecast horizons. For brevity, forecasting results (96 in total) are shown for **Day 13** only, where out-of-sample forecasts are generated using all the preceding data (Day1–Day12) for training. The comprehensive set of results ranging over the duration of study is presented in Table 3.

#### 3.2.1. Seasonal Autoregressive Integrated Moving Average (SARIMA)

**Figure 5:**
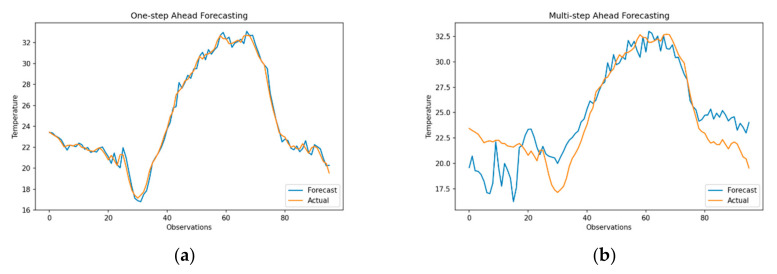

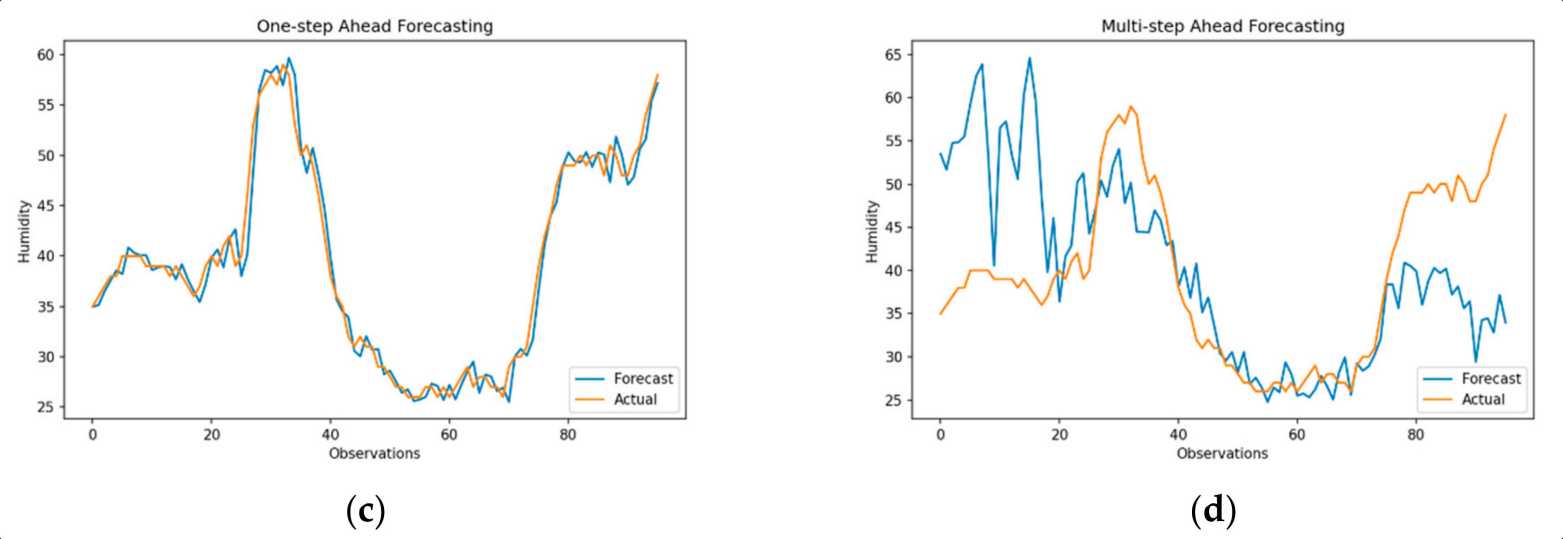
Forecast using Seasonal Auto-Regressive Integrated Moving Average (SARIMA) (**a**) One-step ahead Temperature (*RMSE* = 0.513) (**b**) Multi-step ahead Temperature (*RMSE* = 3.425) (**c**) One-step ahead Humidity (*RMSE* = 2.034) (**d**) Multi-step ahead Humidity (*RMSE* = 10.219) (*top-left, top-right, bottom-left, bottom-right*).

The seasonal ARIMA class of models is widely used for time series forecasting for its ability to effectively model the data distribution, capture trend and seasonality of data and identify best-fitting models using the Box–Jenkins approach [51]. Figure 5 shows the results for one-step and multi-step ahead forecasts generated using the SARIMA (3,1,3) (0,1,0)_96_ model, where 96 represents the frequency of data with drift. In addition, to identifying a suitable subset of models, the model identified using ACF and PACF plots was compared against model specifications generated by the *autoarima* function. The results show that one-step ahead forecasts for temperature (top-left) and humidity (bottom-left), arguably overfit the data with an RMSE of 0.51 (sMAPE of 1.35) and RMSE of 2.03 (sMAPE of 3.62), respectively. One-step ahead forecasts in *R’s forecast* package are generated by defining the forecast horizon with values refitted over training data. This *refit* function uses the model order identified in the previous step. Similarly, multi-step ahead forecasts are generated by specifying the forecast horizon with *predict* function, which implicitly uses a recursive one-step ahead forecast to achieve the desired forecast horizon.

In contrast to one-step ahead forecasting, multi-step ahead forecasts (Figure 5b,d) provide a more realistic representation of forecast performance of SARIMA model over a prediction horizon of 96 observations with an RMSE of 3.425 (sMAPE = 12.14) for temperature and an RMSE of 10.21 (sMAPE = 20.312) for humidity. sMAPE values show that although the model efficiently captures the data’s seasonal behavior, forecasting humidity is more challenging due to the larger variance associated with previous observations. Moreover, as SARIMA models are susceptible to change in the mean, they are often used in conjunction with a smoothing method to suppress sporadic changes in forecasts. Although the forecasts for temperature and humidity are slightly analogous, RMSE values for humidity are much larger, as negative errors are largely penalized during estimation of mean squared error.

#### 3.2.2. Support Vector Regression (SVR)

**Figure 6:**
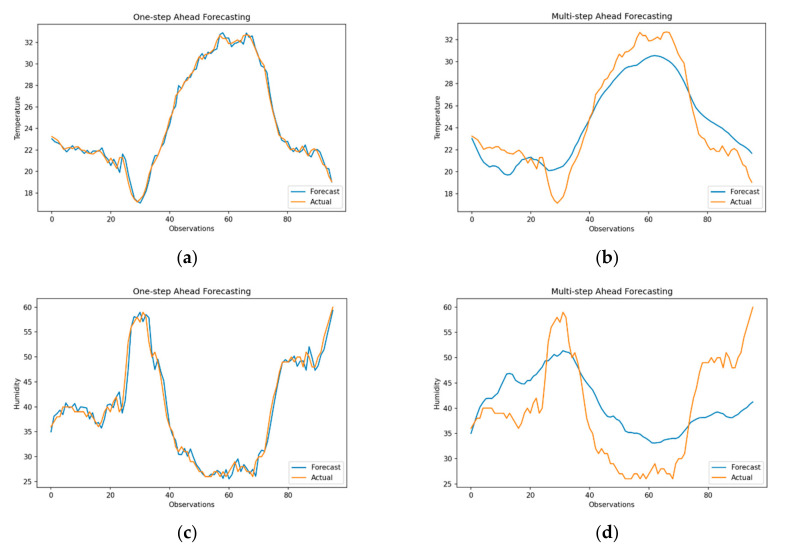
Forecast using Support Vector Regression (SVR) (**a**) One-step ahead Temperature (*RMSE* = 0.478) (**b**) Multi-step ahead Temperature (*RMSE* = 3.141) (**c**) One-step ahead Humidity (*RMSE* = 2.168) (**d**) Multi-step ahead Humidity (*RMSE* = 9.556) (*top-left, top-right, bottom-left, bottom-right*).

Unlike traditional autoregressive models that employ a parametric approach to predictive modelling of data distribution, support vector regression relies on parametric modelling of data using either a linear on a non-linear RBF kernel for reprojecting the observations into a higher dimensional feature space with an epsilon sensitive decision boundary [59]. Additionally, as SVR requires data to be labelled, the training and testing datasets are converted into supervised learning problems by generating sliding windows of size 96, where each feature within the window is a self-lagged variable of timeseries. Furthermore, feature scaling is applied before applying the RBF kernel, where the hyperparameters C = 1.0, gamma = 0.01 and epsilon = 0.2 are selected using a grid search. These values are kept constant over each forecast iteration to standardize cross-validation of data. Forecasting results for one-step vs. multi-step ahead methods for SVR are shown in Figure 6. In line with the results of SARIMA, one-step ahead forecasts seem to overfit the out-of-sample data with RMSE of 0.47 (sMAPE = 1.21) for temperature and RMSE of 2.16 (sMAPE = 3.62) for humidity.

However, for multi-step forecasts, SVR preserves the seasonality and normalizes any rapid fluctuations in data. This is mainly due to the regularization around permissible values of epsilon that minimizes the mean squared error. As a result, the temperature forecasts closely resemble the test data with an RMSE of 3.14 (sMAPE = 12.0). Although for humidity, high and low variations are removed, and the forecasts are marginally accurate with and RMSE of 9.55 (sMAPE = 15.28), while following the trend in data. It is important to note that SVR generates only *one output* (forecast) at each forecast iteration, instead of a series, as in the case of autoregressive models.

#### 3.2.3. Multilayer Perceptron (MLP)

Multilayer Perceptron with backpropagation is widely used for time series forecasting for their ability to learn the model parameters during training, instead of making prior assumptions over data distribution or separation boundaries through reprojection. Before model evaluation, the data were first converted into a supervised problem (similar to SVR) with lagged variables as timeseries features. Several model configurations were evaluated by varying the hyperparameters such as the number of hidden layers, number of neurons in hidden layers, activation function, number of epochs and loss metrics to optimize the model definition and selection. As discussed before, the MLP model with 192 nodes in the hidden layer (rounded off to multiples of the sampling frequency) and one output node was finally chosen, where the number of nodes is equal to 2N + 1, for N as the forecast horizon. The model was trained for 500 epochs with the “ReLU” for node activation, an ADAM optimizer for gradient descent and mean squared error for the error metric.

**Figure 7:**
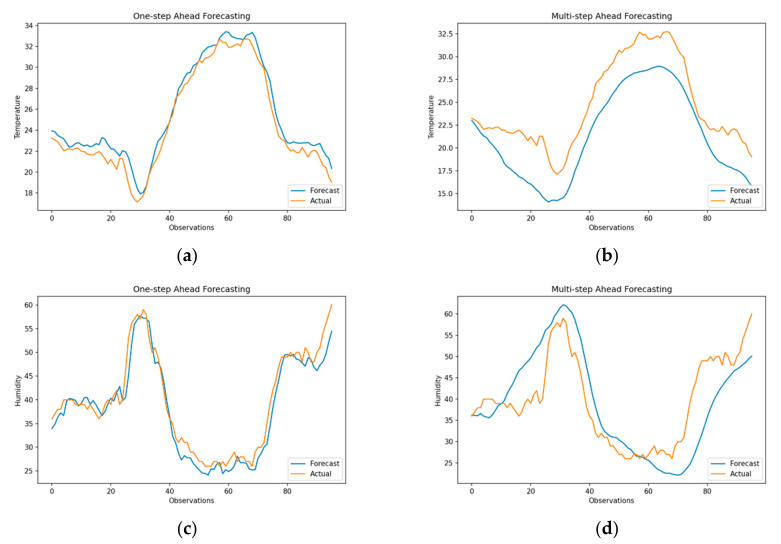
Forecast using Multilayer Perceptron (MLP) (**a**) One-step ahead Temperature (*RMSE* = 1.141) (**b**) Multi-step ahead Temperature (*RMSE* = 7.513) (**c**) One-step ahead Humidity (*RMSE* = 2.873) (**d**) Multi-step ahead Humidity (*RMSE* = 14.486) (*top-left, top-right, bottom-left, bottom-right*).

Results for one-step ahead forecasting using MLP are shown in Figure 7a,c, where model forecasts are closely related to out-of-sample test data with an RMSE of 1.14 (sMAPE = 3.95) and 2.87 (sMAPE = 5.66) for temperature and humidity, respectively. However, it can be observed that forecasts are slightly shifted in time when compared to forecasts from SRIMA and SVR. This small variation is due to the persistent model loss over training epochs, where updates in model weights saturate in the absence of a priori information. On the contrary, multi-step forecasts perform poorly with RMSE of 7.51 (sMAPE = 34.64) and 14.48 (sMAPE = 42.96) for temperature and humidity, respectively. It was also observed that even with the increase in the number of hidden layers, the performance of multi-step ahead forecasts did not necessarily improve, indicating the limited learning capability of the model [54].

#### 3.2.4. Recurrent Neural Networks (RNN)

Multilayer Perceptron often performs poorly over long forecast horizons due to the lack of temporal feedback during model training. Due to this, MLPs cannot model the long-term interdependencies between observations such as seasonality and autocorrelation between variables. To accommodate the temporal variability in data, different altercations of vanilla RNN, including Multi-layered RNN layers with fully connected dense MLP hidden layers (RNN–Dense–Dense) and Stacked RNNs (RNN–RNN–Dense–Dense) were evaluated during experimentation. It was observed that stacked RNN architecture was highly susceptible to vanishing gradients for the univariate dataset used for this study. Finally, a multi-layer RNN model with 192 RNN cells (multiple of sampling frequency) and a fully connected dense layer was trained for 500 epochs using “ReLU” activation for RNN cells and an ADAM optimizer while minimizing mean squared error on forecasts.

**Figure 8:**
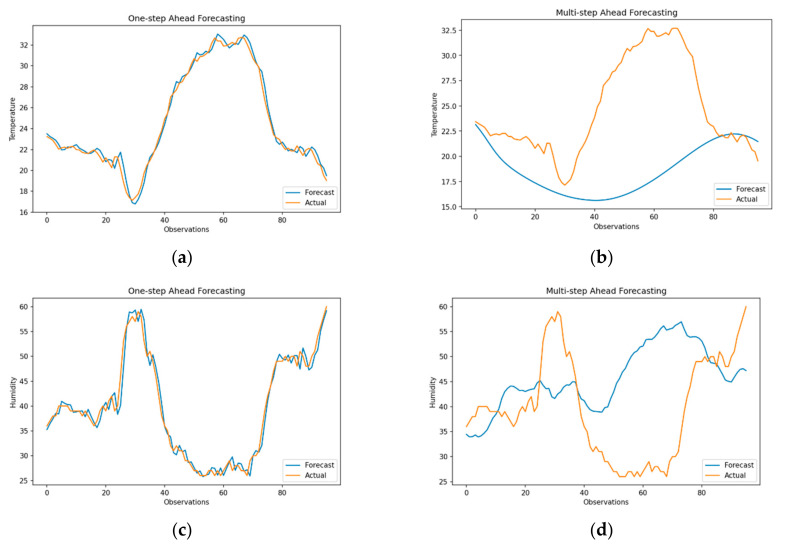
Forecast using Recurrent Neural Networks (RNN) (**a**) One-step ahead Temperature (*RMSE* = 0.653) (**b**) Multi-step ahead Temperature (*RMSE* = 5.581) (**c**) One-step ahead Humidity (*RMSE* = 2.508) (**d**) Multi-step ahead Humidity (*RMSE* = 27.703) (*top-left, top-right, bottom-left, bottom-right*).

As shown in Figure 8a,c, results of the RNN model for one-step ahead forecasts are identical to those of the SARIMA, SVR and MLP models with an RMSE of 0.65 (sMAPE = 1.92) and 2.50 (sMAPE = 4.30) for temperature and humidity, respectively. On the other hand, the performance RNN on multi-step ahead forecasting of temperature is poor with an RMSE of 5.58 (sMAPE = 13.26), albeit the model does replicate the seasonality of data. Additionally, multi-step ahead forecasts for humidity are also extremely poor with RMSE of 27.70 (sMAPE = 51.45). It is important to note that RNNs are designed to model the temporal dependencies of data but are particularly sensitive to high variance (SD = 17.61) and heteroskedasticity in data. As backpropagation in time approximates error over an unrolled structure of RNN and accumulates error over time, any sudden changes in intermediate derivate can adversely impact the performance of RNN over a longer forecast horizon. Additionally, RNN models can generate the sequence as output (seq2seq models) based on model configuration and application requirement. However, multi-layered RNN architecture adopted for this study generates *one output* at each iteration, thereby severely limiting the forecasting capabilities of the model. Nonetheless, this is important for substantiating the comparison between single-output (SARIMA and SVR) and multi-output models (RNN and LSTM) and normalizing any inherent bias in forecasts.

#### 3.2.5. Long-Short Term Memory (LSTM)

As discussed before, Long-Short Term Memory are highly efficient in modelling long temporal dependencies in data without the pitfall of exploding or vanishing gradients. LSTMs can explicitly control memory cells’ current and previous state by using control gates for information retention. Due to this, training LSTMs is more straightforward (and slower due to extra cell states) but requires a large volume of training data. Analogous to previous approaches, data were first converted into a supervised learning problem using a sliding window-based approach and feature scaling is applied before inputting the data into the LSTM model [60]. Analogous to RNN modelling, different LSTM architectures, including multilayer LSTMs (LSTM–Dense–Dense) and stacked LSTMs (LSTM-LSTM–Dense–Dense) were evaluated to identify the suitable sequential model. It is important to outline that choosing layer configurations, model parameterization and hyperparameter tuning in sequence models is often a trial-based approach and aims at reducing the model error over training epochs. Thus, there is no best model which can achieve reproducible performance over different temporal epochs of timeseries data. Based on training accuracy and forecast performance, a model with 96 LSTM cells followed by a Dense layer with 12 neurons in the hidden layer and an output layer with a single neuron is used with a “ReLU” activation function and an ADAM optimizer was selected. The model was trained for 500 epochs using mean square error as the accuracy metric.

**Figure 9:**
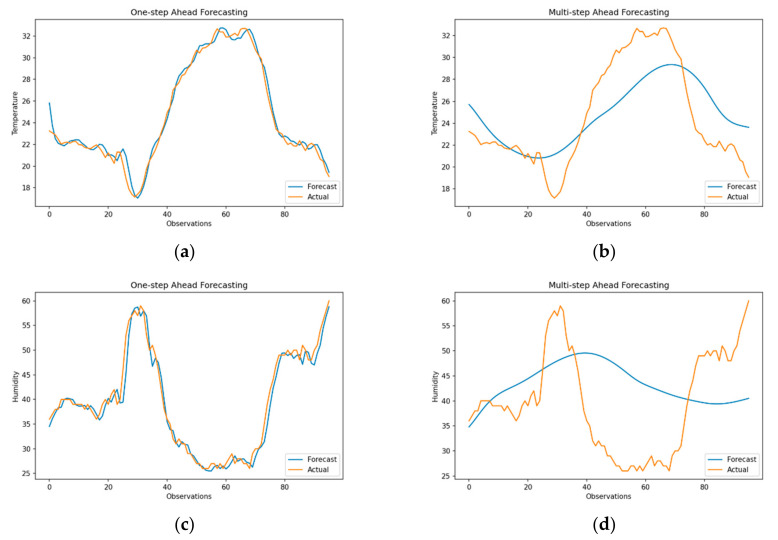
Forecast using Long-Short-Term-Memory (LSTM) (**a**) One-step ahead Temperature (*RMSE* = 0.585) (**b**) Multi-step ahead Temperature (*RMSE* = 2.807) (**c**) One-step ahead Humidity (*RMSE* = 2.117) (**d**) Multi-step ahead Humidity (*RMSE* = 8.087) (*top-left, top-right, bottom-left, bottom-right*).

Results for the forecasting performance of the one-step ahead LSTM TSFA are shown in Figure 9a,c. As evident from previous results, one-step ahead forecasts efficiently replicate the out-of-sample test data with an RMSE of 0.58 (sMAPE = 1.41) and 2.11 (sMAPE = 3.44) for temperature and humidity time series. On the contrary, results for multi-step ahead forecasts highlight the inability of the model to make long term forecasts in the current settings, where the model is forced to generate single-output at each forecast iteration. Moreover, results for temperature with RMSE of 2.80 (sMAPE = 8.67) and humidity with RMSE of 8.08 (sMAPE = 17.51) in the multi-step ahead setting suggests that the model performance is comparable with SARIMA and SVR models, as shown in Table 3. This underlying contradiction between the value of the error matric and forecasts is due to two significant factors. Firstly, LSTMs perform exceptionally well for complex timeseries datasets, where long-term dependencies play a crucial role in maintaining intermediate cell state and model training. Due to this, from simpler timeseries, the internal cell structure of LSTM has “very little” information to retain, thereby adversely affecting the model performance with limited data. Secondly, to enhance the forecasting performance, LSTMs rely on selective feature engineering using intermediate layers of convolution (ConvLSTM) and batch normalization to select prominent data features. In the absence of such architecture, LSTMs tend to underperform when compared against more robust statistical models and generalize the forecasts based on the data’s underlying pattern.

### 3.3. Performance Evaluation of TSFAs over One-Step and Multi-Step Ahead Forecast Horizon

**Figure 10:**
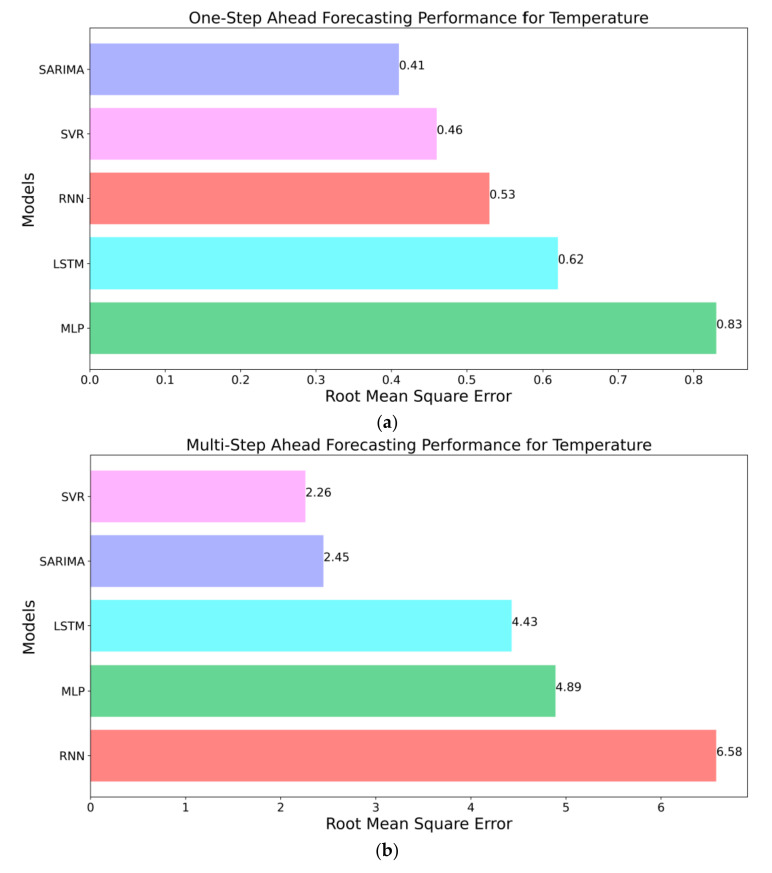

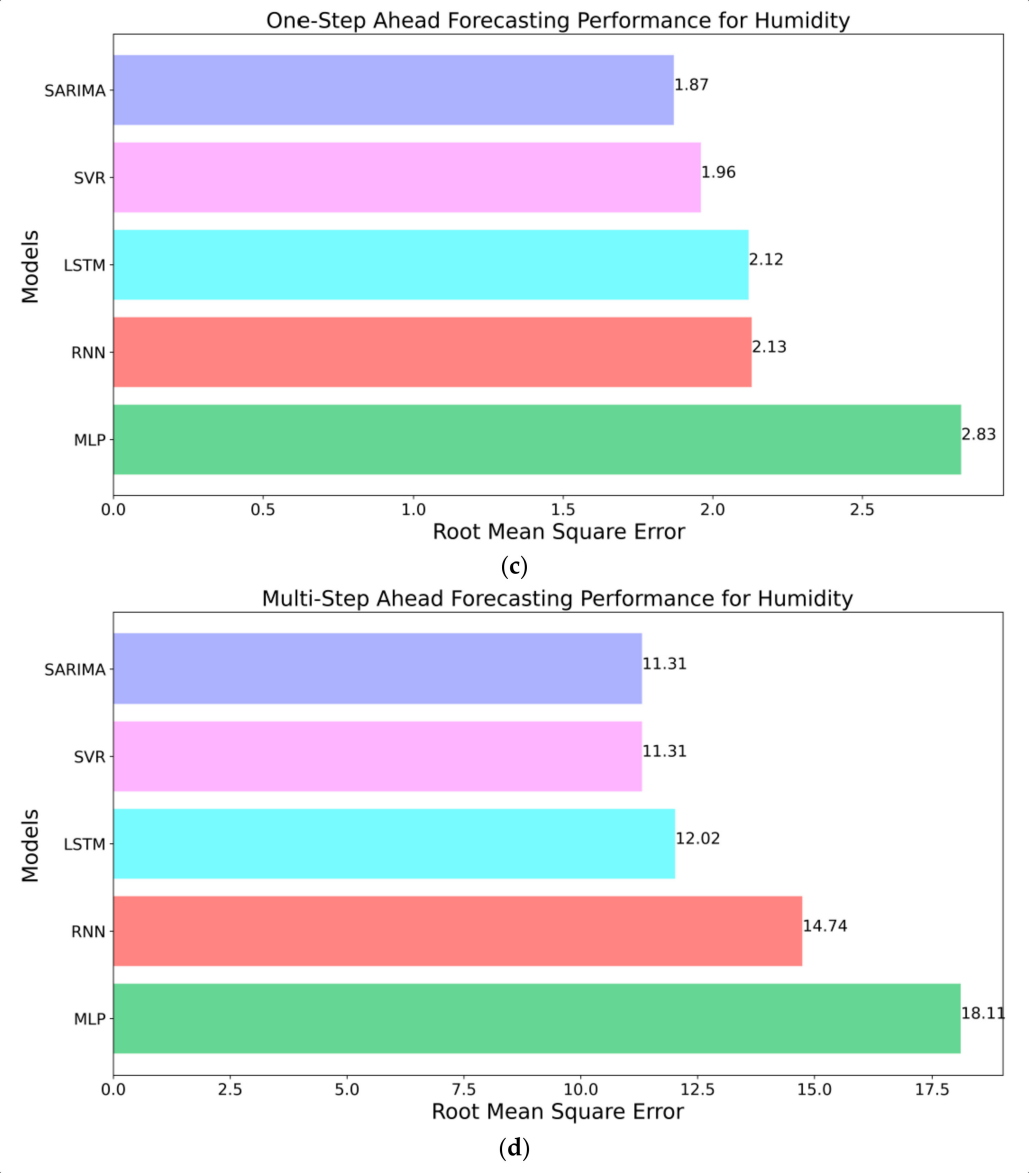
Best performing models and associated RMSE values for (**a**) One-step ahead Temperature (**b**) Multi-step ahead Temperature (**c**) One-step ahead Humidity (**d**) Multi-step ahead Humidity (*top-to-bottom*).

It is evident from previous results that the forecasting ability of TSFAs over unseen data can be evaluated by approximating the Root Mean Squared Error (RMSE) between out-of-sample forecasts and actual observations using expanding windows and walk-forward validation. In this section, Figure 10a–d present cumulative forecast errors of TSFAs over one month and characterize the models’ generalized performance for multi-horizon forecasts. Here, RMSE values for each TSFA are plotted in sorted order with best performing model at the top. Figure 10a suggests that SARIMA was the best model for one-step ahead forecasting of temperature timeseries, with an RMSE of 0.41 closely followed by SVR with an RMSE of 0.46. It is evident that MLP was the worst-performing model with an RMSE of 0.83 due to its inability to model temporal dependencies and resemblance to the naïve forecast. Figure 10b for multi-step ahead forecasting of temperature also exhibits a similar trend with SVR as the best performing model with an RMSE of 2.26 followed by the SARIMA model (RMSE of 2.45). In this, LSTM outperforms RNN and MLP models with an RMSE of 4.43, given its superiority in retaining information long-term. In both cases, it can be seen that statistical models outperform recurrent models (RNN and LSTM) by a large margin.

The forecasting performance of TSFAs over humidity time series is consistent with results for temperature forecasting with added variability due to the highly stochastic nature of timeseries data. Figure 10c suggests that SARIMA is the best performing model for one-step ahead forecasting with an RMSE of 1.87 followed by SVR with RMSE of 1.96. Furthermore, RNN and LSTM perform moderately well with RMSEs of 2.12 and 2.13, respectively. For multi-step ahead forecasting (Figure 10d), SARIMA and SVR perform equally well with RMSE of 11.31. In both the cases (one-step vs. multi-step), RNN (RMSE 2.13 and 14.74) and LSTM (RMSE 2.12 and 12.02) perform significantly better than MLP due to the inherent recurrent architecture. Finally, it is inferred that with limited training data, statistical models can outperform neural models with moderate forecast horizons. The results presented in this section are in line with observations and conclusions made by a few other studies, which serve as an excellent benchmark for time series modelling and forecasting competitions [14,49,61,62].

### 3.4. Assessing Impact of Data Bifurcation on Forecasting Capability of TSFAs

**Figure 11:**
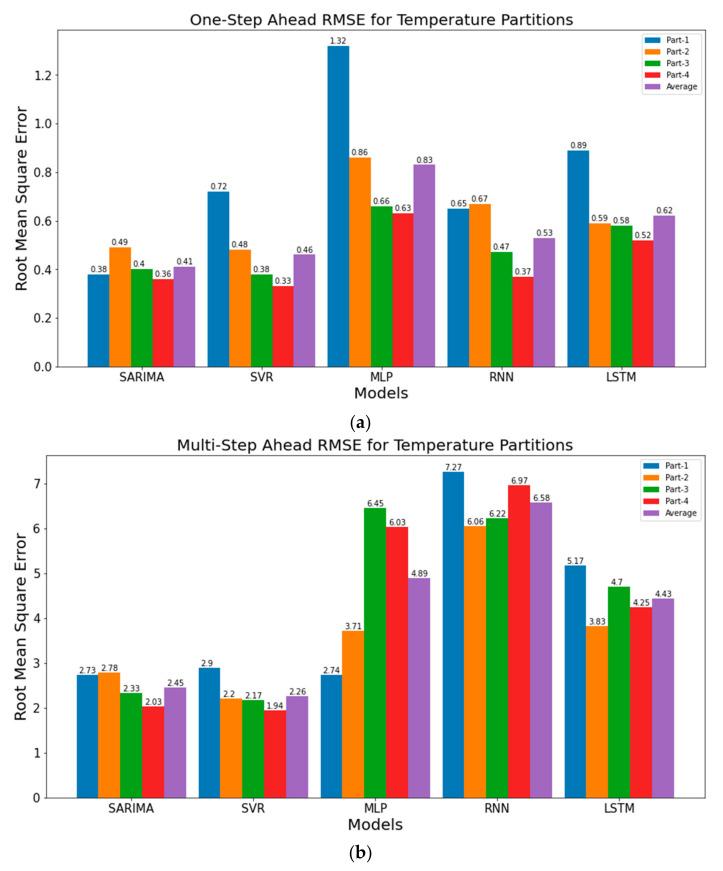

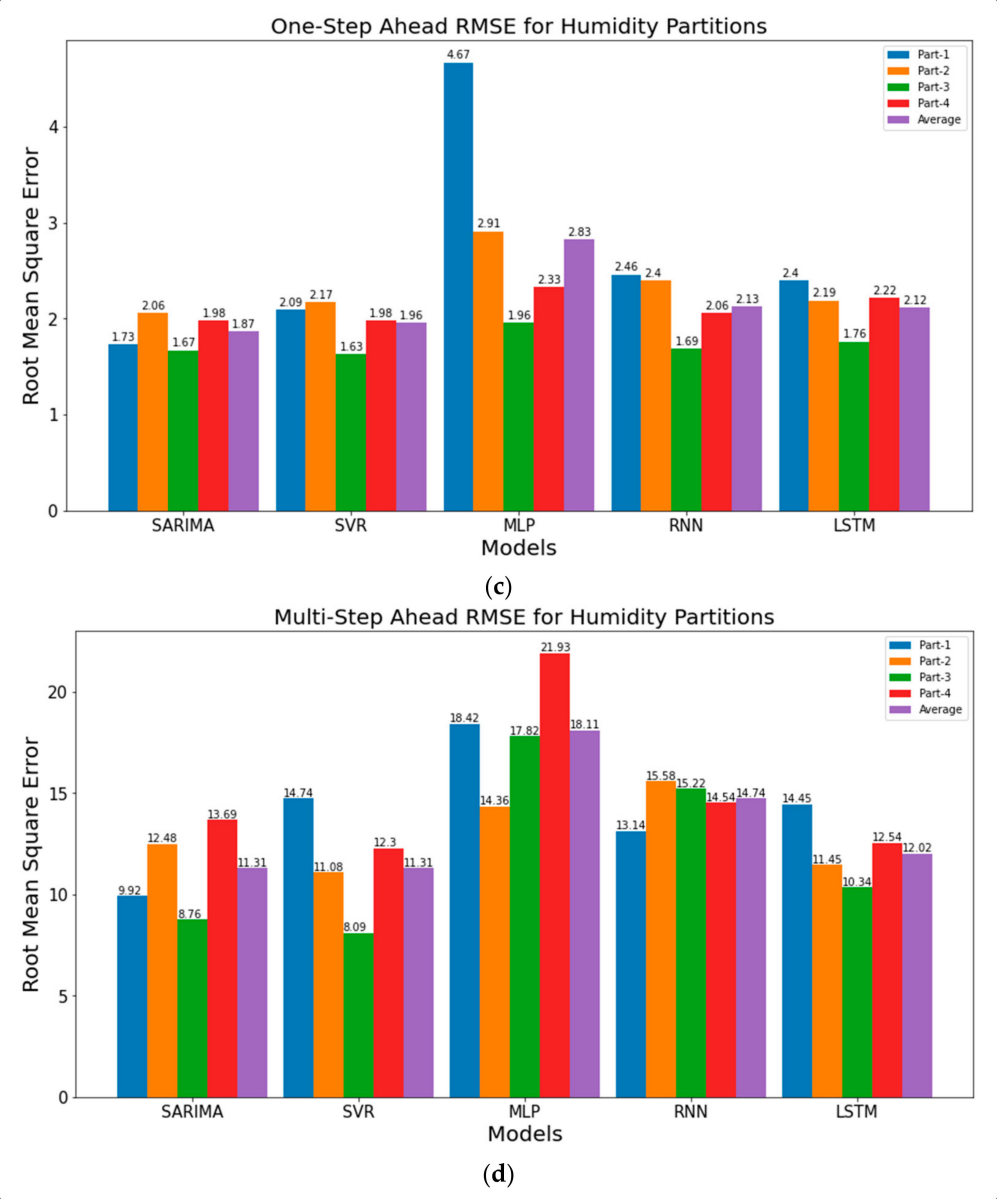
Comparison of forecast performance of TSFA over fixed data partitions (7-day) and average performance with walk-forward validation (**a**) One-step ahead Temperature (**b**) Multi-step ahead Temperature (**c**) One-step ahead Humidity (**d**) Multi-step ahead Humidity (*top to bottom*).

As discussed before, partitioning of the timeseries dataset is crucial for assessing the performance of TSFAs over a long forecast horizon [16]. With walk-forward validation, the models are re-evaluated over a specific time period, defined by the expanding window (96 in this study). In this, all observations preceding the current window size are used as training data, while the contents of the present window are used as the testing dataset. However, this approach can result in unreliable results when the dataset has significant temporal variations in data distribution for each sub-partition (Table 1). Under these circumstances, the performance of TSFAs is evaluated using (a) average performance over entire dataset (b) performance over each sub-partition of data. The results for RMSE of models over different partitions are shown in Figure 11a–d where Part1 consists of observations from Day1 to Day7 (P1), Part 2 is from Day8 to Day14 (P2), Part 3 is from Day15 to Day21 (P3) and Part 4 is from Day22 to Day28 (P4). Details for each partition and naming conventions are detailed in Table 2. It is important to note that the values for RMSE are averaged over the above partitions, instead of evaluating the model over specific partitions. This averaging is crucial for recurrent models that tend to underperform with minimal data (7 × 96 observations in this case).

Figure 11a shows RMSE values for one-step ahead forecasting of temperature, where the performance of TSFAs over P1 is extremely poor compared to the “Average” model performance. This is due to high modelling error during the initial stage modelling with a limited number of observations. With time and the expanding window of training, the performance substantially improves over partitions 3 and 4, which is still lower than the average. This has a dual impact on how the results for forecasting are interpreted while using walk-forward validation. Firstly, the average performance is not a representative approximation of model performance over temporal partitions, where the results can either be underestimated or overestimated in time. For example, RMSE for SVR over Part1 is 0.72, while it drops to 0.33 over Part 3. However, the average performance of the model stalls at RMSE of 0.46. This phenomenon is observed for all the models, except for SARIMA where the initial error over Part 1 (RMSE = 0.38) is lower than the overall error of 0.41. Such partition-based analysis serves as a baseline for transfer learning and deploying pre-trained time series models onto IoT-based Edge devices. Under such circumstances, the decision to re-evaluate the model is based on the error threshold obtained using current temporal error and pre-evaluated average error approximated during training, thus forcing unnecessary model re-training. Secondly, the average performance does not provide any insights into the model regression error resulting from drastic changes in recent observations. Events such as sensor malfunctioning or rapid change in observed phenomena, like rainfall in summers (outliers), can create unreliable forecasts by averaging the temporal errors. Similar conclusions can be drawn from Figure 11b–d, especially for one-step ahead performance of humidity, where RMSE of models over Part 1 and 2 suppresses the model performance over partition 3, thereby increasing the RMSE on the overall dataset (average). Another important inference drawn from the multi-step ahead performance of the timeseries (Figure 11b,d) is that increasing the size of the training set (P4 includes all the observations until P3 and the extended window) with recent observations does not necessary improve the performance of TSFA over the long forecast horizon.

### 3.5. Visualization for Absolute Performance of TSFAs Using Baseline Naïve and Seasonal Naïve Methods

**Figure 12:**
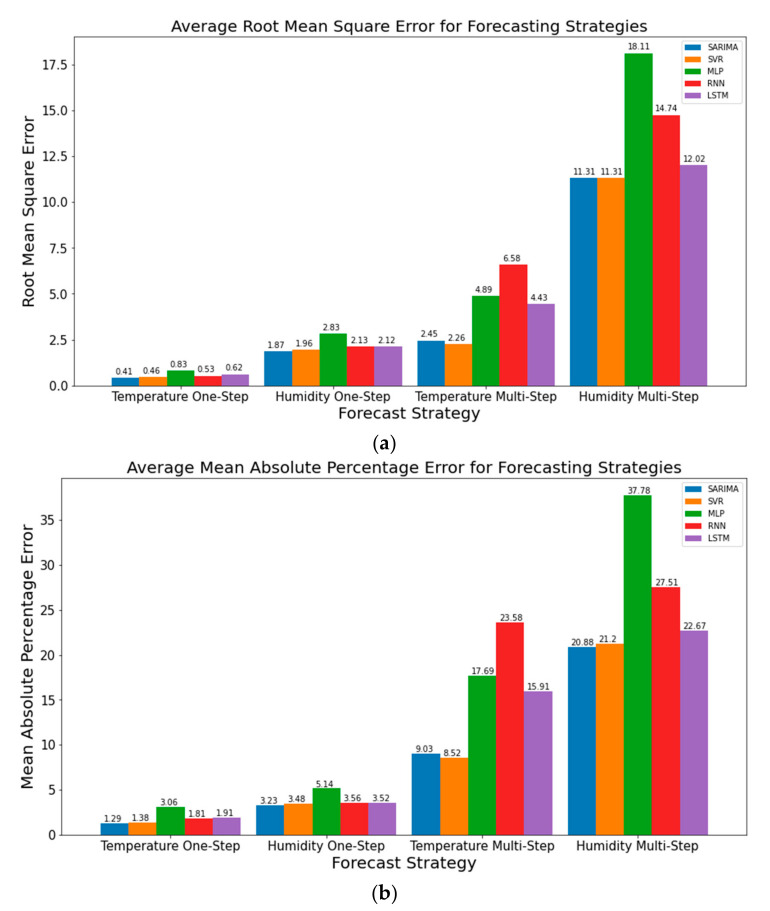
Average performance for forecasting strategies over one-step and multi-step ahead forecasts (**a**) Root Mean Squared Error (**b**) Symmetric Mean Absolute Percentage Error.

Results presented in previous subsections demonstrate the practicality and challenges of using TSFAs to make one-step and multi-step ahead forecasts. It also shows a thorough comparison between important and widely used TSFAs while establishing a premise for using data bifurcation schemes to correctly interpret the model performance in terms of accuracy metrics such as Root Mean Squared Error. However, it is often observed that model forecasts are biased and mimic the behavior of the original time series, offset by a white-noise-based error component. In simple terms, the forecasts are a time-shifted variant of the original time series. Under such circumstances, it is crucial to assess the performance of TSFAs against a simple baseline model and observe the direction of prediction in the following timeframe instead of focusing on quantitative assessment through error metrics. This approach simplifies the task of model interpretation and evaluating the model performance in absolute terms. For this, the first approach uses Symmetric Mean Absolute Percentage Error (sMAPE) to assess the forecast performance of models over different forecast horizons. sMAPE is often used for intercomparison of models in absolute terms. The symmetric component helps establish an upper and lower bound on model performance, thereby preventing under or overestimation of forecasts. The results presented in Figure 12 (unified image for Figure 11), show that the average performance of models using RMSE is similar to that of sMAPE, over the one-step and multi-step ahead forecast horizon. Although both RMSE and sMAPE are acceptable performance metrics for assessing model performance, RMSE is more commonly used in academic literature.

**Figure 13:**
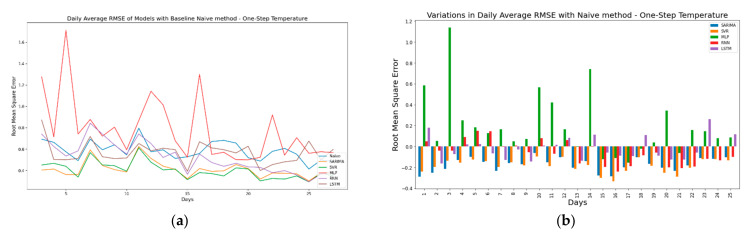

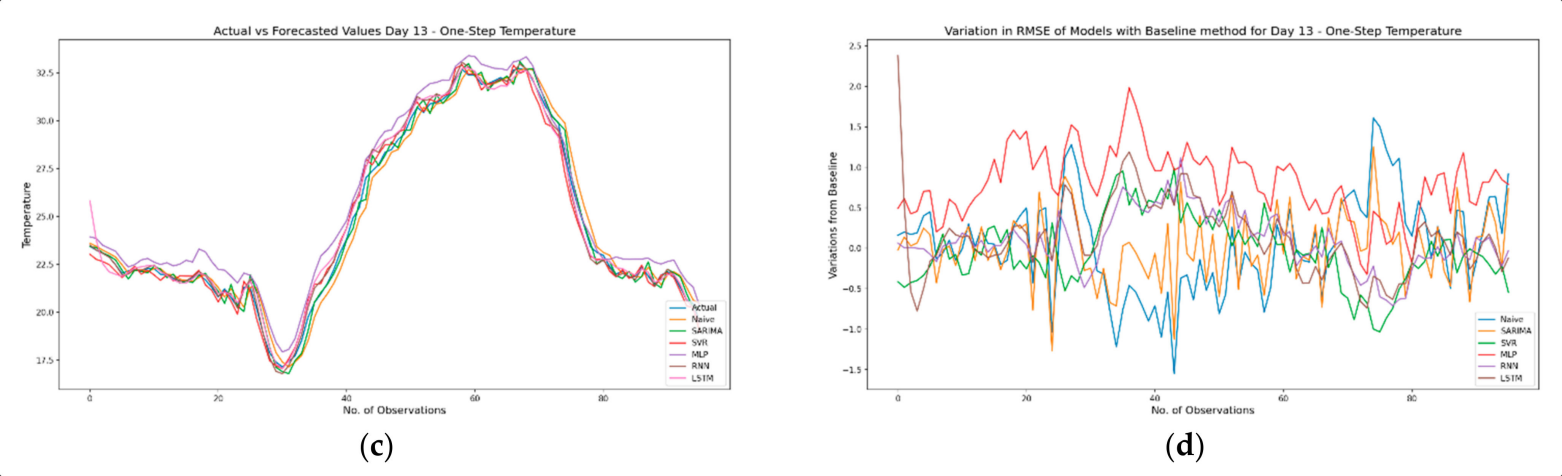
Comparative visualization for model performance against a baseline model—One-step ahead Temperature (**a**) daily average RMSE of different models with a baseline naïve model (**b**) variations in daily average RMSE with performance against naïve model (**c**) combined Actual vs. Forecasts generated by different TSFAs for Day 13 (**d**) variations in RMSE of models against naïve model for Day 13.

As discussed before, the second approach for evaluating model performance is to compare the model forecasts against the forecasts generated by a baseline model. For the one-step ahead forecast horizon, a simple naïve model was used. Here, the naïve model is a one-step time-shifted variant of the original time series. In simple terms, the forecasts at time t are given by $f_{t+1}=\;f_{t}$. For the multi-step ahead forecast horizon, a seasonal naïve model was used where the forecasts for the current timeframe are actual observations from the previous seasonal timeframe or, simply put, the original time series is seasonally shifted in time. Figure 13a shows the daily averaged RMSE values for one-step ahead Temperature forecasts compared against a naïve model. Figure 13b shows the variations in RMSE values against the naïve model. Here, the negative values represent that the forecasts from TSFAs are better than the baseline model. Finally, Figure 13c,d presents the actual vs. forecasted values for Day 13 and variations between values forecasted by TSFAs and the naïve model. The results confirm that the forecasts generated by TSFAs are not biased or time-shifted and are reliable for one-step ahead forecasting. It also shows that all TSFAs perform better than the baseline model except for MLPs with a poor forecast performance across all days.

**Figure 14:**
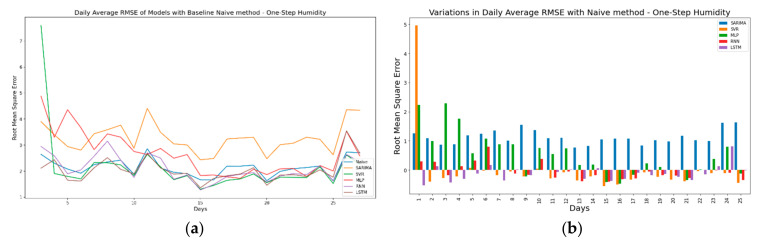

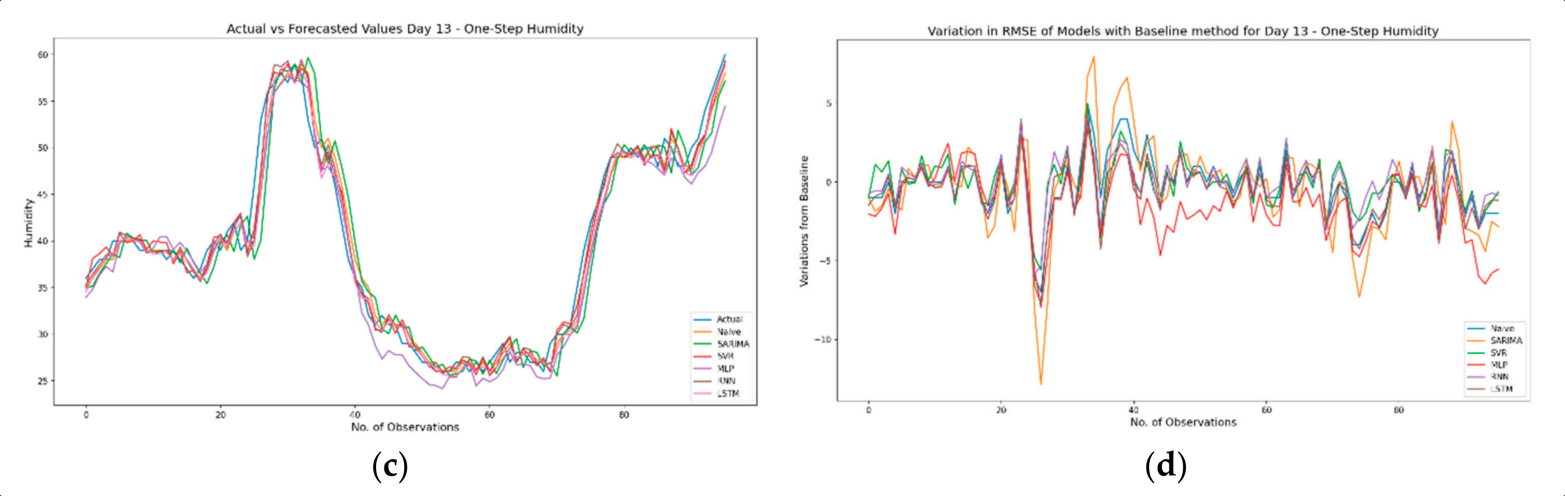
Comparative visualization for model performance against a baseline model—One-step ahead Humidity (**a**) daily average RMSE of different models with the baseline naïve model (**b**) variations in daily average RMSE with performance against naïve model (**c**) combined Actual vs. Forecasts generated by different TSFAs for Day 13 (**d**) variations in RMSE of models against the naïve model for Day 13.

In continuation, Figure 14a shows the pattern of daily average RMSE along with the baseline naïve model for one-step ahead humidity. Similar to Figure 13a, the values for daily average RMSE are coherent and closely related in time. This is expected, as for one-step ahead forecasts only one value is forecasted ahead in time. Additionally, it is interesting to note that variations in daily average RMSE values for one-step ahead temperature (Figure 13b) are more stable when compared against values for one-step ahead humidity (Figure 14b), mainly due to high variability in humidity observations during a small temporal window. It also shows that for both one-step ahead humidity and temperature, models show positive deviation from the naïve method (more positive bars in Figure 14b), which suggests that the performance of TSFA models is below par at multiple time steps. Generally, this indicates poor model selection and forecasting capabilities for TSFAs. However, this does not hold valid for high-frequency seasonal time series where the observations are closely placed in time and are highly correlated. Due to this temporal correlation, the naïve model with a one-step ahead forecast horizon tends to outperform most time series models that rely on time series features to generate forecasts [63].

**Figure 15:**
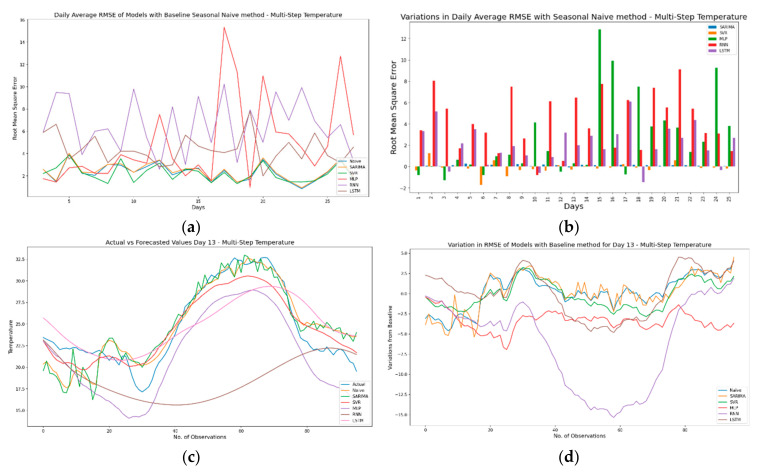
Comparative visualization for model performance against a baseline model—Multi-step ahead Temperature (**a**) daily average RMSE of different models with baseline seasonal naïve model (**b**) variations in daily average RMSE with performance against seasonal naïve model (**c**) combined Actual vs. Forecasts generated by different TSFAs for Day 13 (**d**) variations in RMSE of models against seasonal naïve model for Day 13.

**Figure 16:**
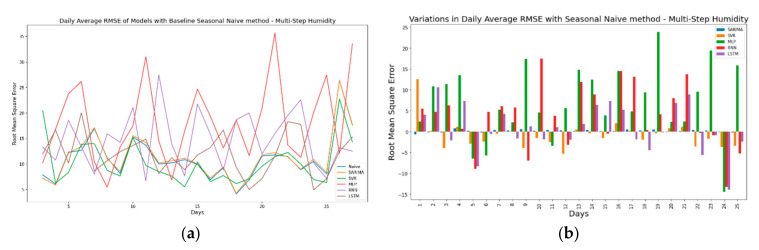

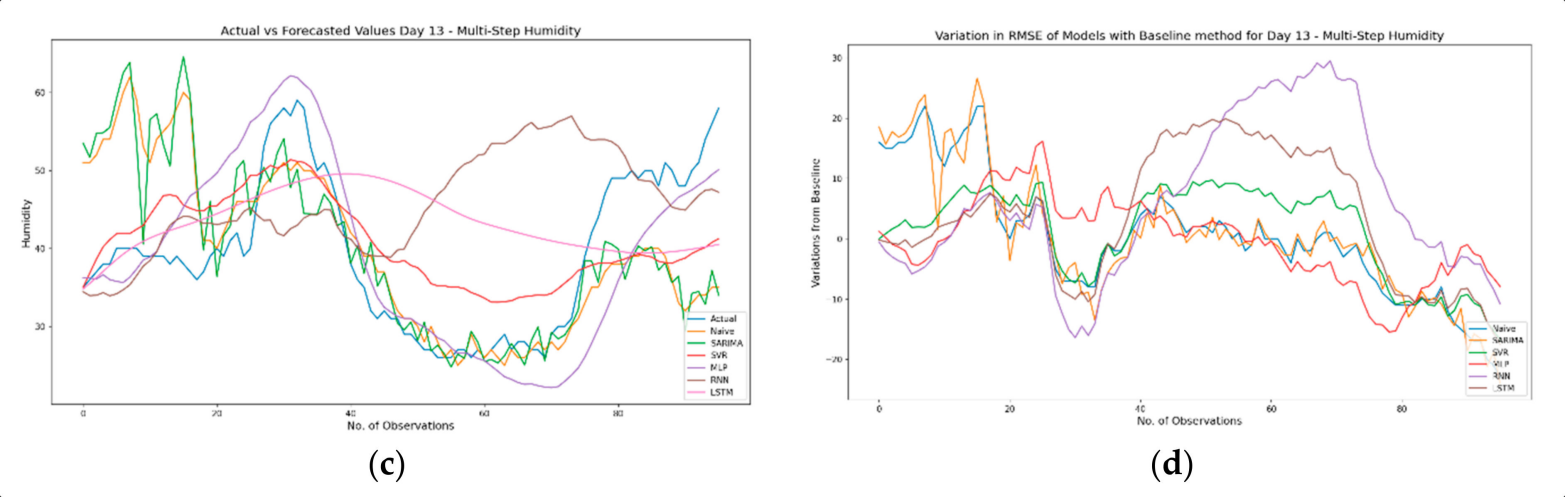
Comparative visualization for model performance against a baseline model—Multi-step ahead Humidity (**a**) daily average RMSE of different models with baseline seasonal naïve model (**b**) variations in daily average RMSE with performance against seasonal naïve model (**c**) combined Actual vs. Forecasts generated by different TSFAs for Day 13 (**d**) variations in RMSE of models against seasonal naïve model for Day 13.

As discussed previously, Figure 15a–d and Figure 16a–d present results for daily variation in RMSE against a baseline seasonal naïve model using multi-step ahead temperature and humidity forecasting strategies. Figure 15a shows the pattern for daily average RMSE along with the baseline seasonal naïve model for the multi-step ahead temperature time series. It is evident that for multi-step ahead forecasts, MLP and RNN are the worst-performing models with high fluctuations in RMSE. Similarly, Figure 15b shows that almost all models have a below-par forecast performance when compared against the seasonal naïve model (significant positive peaks). Additionally, Figure 15c,d confirm that forecasts generated using RNNs show large deviations and are the worst-performing models for multi-step ahead forecasts.

In continuation, Figure 16a shows the pattern of daily average RMSE along with the baseline seasonal naïve model for the multi-step ahead humidity time series. Here, again, RNNs and MLP are outperformed by other TSFAs by a large margin. However, Figure 16b,d show an interesting pattern where TSFAs occasionally outperform the seasonal naïve model. This clearly indicates the presence of structural breaks in the time series, where high variability forces the naïve model to perform better over certain timesteps and vice versa. Figure 16c presents another interesting insight where SARIMA tries to mimic the seasonal naïve model instead of producing unbiased forecasts. This behavior can be attributed to the structure of autoregressive models which are based on modelling seasonal components and the distribution of time series, very similar to a seasonal naïve model.

The results presented in this subsection suggest that assessing the model performance against a baseline model provides valuable insights into the direction of the forecast and simplifies the task of forecast interpretation over unseen data. However, it is also important to critically analyze the underlying methodology for model selection and forecast generation. This is especially true for results that show that a naïve model can outperform a standard TSFA, which is typically true for a low-frequency time series. On the contrary, as consecutive observations of high-frequency time series (diurnal) have high temporal correlation, a simple time-shifted naïve model can outperform most TSFAs. Similarly, for a multi-step ahead forecast horizon, if the forecasts are generated using the iterative strategy (as used in this work), the errors after each forecast iteration are accumulated over the forecast window. Due to this, a simple seasonal naïve model, which does not account for error accumulation, can outperform other TSFAs by large margins. Finally, to generate absolute benchmarks, other multi-step forecast generation methods, such as direct or multi-input–multi-output, and other benchmark methods such as rolling mean or STL-based benchmarking should be further explored.

## 4. Conclusions and Future Prospects

Uninterrupted monitoring of agrometeorological parameters is quintessential in Precision Agriculture (PA), where the accuracy of ahead-of-time predictions can significantly impact decision-making and management. For this, state-of-the-art sensing platforms, such as Edge and Fog-based architectures, play a pivotal role in enabling on-field data processing and information generation through real-time data assimilation. When augmented with pre-trained Time-Series Forecasting Algorithms (TSFAs), these devices can generate real-time multi-step ahead forecasts, enabling on-field analytics. In a similar setting, this work presents an in-depth analysis for forecasting capabilities of popular TSFAs over one-step and multi-step ahead forecast horizons using agrometeorological time series of temperature and humidity over one month. Initially, a methodological approach for recursive estimation of n-step ahead forecasts is presented with due diligence to the importance of using walk-forward validation during model evaluation. Furthermore, the work discusses the rationale behind using data partitions and provides a brief overview of TSFAs employed in this study. Finally, the experimental setup and model-specific configurations are discussed to ensure the reproducibility of results. The results suggest that, in general, statistical models SARIMA and SVR, that are considerate towards data distribution outperformed deep-learning-based models (MLP, RNN and LSTM) which employ iterative minimization and gradient approximation for learning through training samples. One causative factor that explains the presented results is the impaired capability of deep-learning models in forecasting simpler time series. It was also observed that the performance of recurrent models depends on the volume of data and requires extensive tuning for hyperparameters. Evidently, with enough training data and multi-input–multi-output (MIMO) functionality, DL models can outperform statistical models over longer horizons. The results were found to be consistent for both one-step and multi-step ahead forecast methods. This work also outlines the incomprehensible importance of including the n-step forecast horizon while estimating the performance of TSFAs for any application. The results exhibit two extremities of forecasting, where one-step ahead forecasts are inexplicably accurate, while multi-step ahead forecasts quickly become unreliable with an increase in the forecast horizon. Further, a thorough comparison of model performance against a baseline naïve model shows the importance of the forecast direction to make the results more interpretable. Towards the end, the study substantiates the importance of data bifurcation in timeseries, which can help make better forecasts with explainable temporal intervals. However, it is worth mentioning that this study’s approaches are heuristic and essentially require a thorough understanding of the problem statement and the vision behind implementing forecasting infrastructure.

The current scope of work can be extended by evaluating the TSFAs under different configurations and fine-tuning the models using feature engineering. The proposed baseline can be improved by evaluating model performance over multivariate timeseries datasets with global sensitivity analysis to understand the causal relationships in an agrometeorological phenomenon. Furthermore, the results for n-step ahead forecasting can be improved upon by approximating a generalized performance threshold over the expanding forecast horizon, rather than any pre-defined horizon with out-of-sample evaluation. Finally, to understand the impact of temporal performance of TSFAs, it could be thus prospective to substitute the fixed partitioning approach with breakpoint (or structural breaks)-based timeseries analysis.

## Figures and Tables

**Table 1:** Temperature and Humidity Partitions for applying the Kruskal–Wallis and Nemenyi Tests. Values in *italics* denote a significant difference in pair-wise relation while values in bold denote partitions negligible difference.

**Temperature Partitions for KW and Nemenyi Tests**				
	**Temp_0**	**Temp_1**	**Temp_2**	**Temp_3**
**Temp_0**	1	*0.022*	*0*	*0*
**Temp_1**	*0.022*	1	*0*	*0*
**Temp_2**	*0*	*0*	1	**0.943**
**Temp_3**	*0*	*0*	**0.943**	1
**Humidity Partitions for KW and Nemenyi Tests**				
	**Hud_0**	**Hud_1**	**Hud_2**	**Hud_3**
**Hud_0**	1	*0*	**0.654**	**0.086**
**Hud_1**	*0*	1	*0.003*	*0*
**Hud_2**	**0.654**	*0.003*	1	*0.002*
**Hud_3**	**0.086**	*0*	*0.002*	1

**Table 2:** Mean and Standard Deviation of fixed partitions P1, P2, P3 and P4 and average over the entire timeseries for Temperature and Humidity (**bold** and *italic* represent values which are higher and lower than the average mean and SD respectively).

Mean and SD for Data Partitions				
	*Humidity*		*Temperature*	
	Mean	SD	Mean	SD
Day 1–7 (P1)	**48.05**	**18.73**	*22.24*	**6.70**
Day 8–14 (P2)	*42.17*	*13.07*	*23.65*	*5.33*
Day 15–21 (P3)	*45.78*	*15.46*	**26.01**	*5.68*
Day 22–28 (P4)	**51.47**	**20.80**	**26.21**	*4.99*
Total 1–28 (Mean, SD)	**46.88**	**17.61**	**24.52**	**5.94**

**Table 3:** Root Mean Square Error (RMSE) for One-step vs. Multi-step ahead forecasts using Time-Series Forecasting Algorithms (TSFAs) with a forecast horizon of 1-day (96 observations) (*colors represent the scale of values—assigned individually for one-step and multi-step ahead forecasts*).

	One-Step Ahead Forecasting										Multi-Step Ahead Forecasting									
	Temperature					Humidity					Temperature					Humidity				
Day	SARIMA	SVR	MLP	RNN	LSTM	SARIMA	SVR	MLP	RNN	LSTM	SARIMA	SVR	MLP	RNN	LSTM	SARIMA	SVR	MLP	RNN	LSTM
3	0.37	1.88	2.14	0.76	2.06	1.58	2.66	7.12	2.84	4.22	3.20	3.46	4.97	7.59	5.40	10.90	24.49	14.24	8.65	15.48
4	0.40	0.45	1.28	0.74	0.87	2.11	2.39	4.85	2.94	2.05	2.58	2.21	1.74	5.95	5.90	7.24	20.94	10.74	13.64	10.78
5	0.41	0.47	0.72	0.63	0.50	1.81	1.91	3.30	2.68	2.44	1.55	2.72	1.44	9.49	6.63	5.88	6.41	17.26	11.56	16.02
6	0.36	0.44	1.71	0.54	0.50	1.61	1.80	4.35	1.77	1.65	4.03	3.86	2.71	9.39	3.50	12.17	8.49	24.06	18.57	10.22
7	0.36	0.34	0.74	0.58	0.51	1.57	1.70	3.70	2.06	1.62	2.32	2.27	2.86	3.90	4.40	13.43	13.38	25.82	13.26	19.74
8	0.59	0.57	0.88	0.84	0.72	2.14	2.33	2.79	2.57	2.13	2.30	1.84	2.23	6.01	5.55	17.14	14.93	11.05	8.73	8.81
9	0.45	0.45	0.72	0.74	0.53	2.30	2.33	3.43	3.16	2.52	3.04	1.31	2.22	6.22	3.18	11.03	11.29	5.49	16.20	10.81
10	0.41	0.45	0.81	0.64	0.51	2.00	2.25	3.33	2.46	2.10	3.09	3.55	3.90	4.21	4.22	8.57	7.63	12.93	14.16	12.14
11	0.39	0.39	0.59	0.55	0.52	1.69	1.87	2.79	1.78	1.91	2.31	1.40	3.43	9.79	4.22	15.58	15.31	17.93	21.20	13.40
12	0.63	0.61	0.87	0.74	0.65	2.60	2.60	2.56	2.65	2.66	3.04	2.49	3.14	5.45	3.84	14.19	10.85	31.26	7.26	15.34
13	0.51	0.48	1.14	0.65	0.59	2.03	2.17	2.87	2.51	2.12	3.43	3.14	7.51	5.58	2.81	10.22	9.56	14.49	27.70	8.09
14	0.44	0.41	1.01	0.52	0.61	1.68	1.67	2.56	1.68	1.89	2.27	1.68	3.53	8.20	2.98	10.61	8.00	7.38	13.84	11.58
15	0.41	0.41	0.68	0.57	0.60	1.73	1.83	2.60	1.85	1.91	2.62	2.62	2.01	3.01	5.66	11.18	7.52	16.45	7.68	8.62
16	0.32	0.31	0.52	0.36	0.39	1.33	1.31	1.80	1.27	1.36	2.63	2.41	2.99	9.14	4.67	10.03	10.44	24.42	21.69	11.78
17	0.42	0.38	1.30	0.55	0.67	1.52	1.45	1.85	1.49	1.74	1.54	1.38	1.56	4.98	4.26	7.27	6.53	18.90	16.58	13.28
18	0.39	0.37	0.55	0.48	0.61	1.74	1.66	1.79	1.80	1.82	2.59	2.30	15.33	10.23	4.09	9.41	8.95	13.65	9.95	16.62
19	0.40	0.35	0.57	0.44	0.60	1.81	1.71	1.78	1.87	1.90	1.43	1.30	11.35	3.20	4.42	4.27	6.51	18.86	18.70	9.36
20	0.46	0.42	0.50	0.47	0.56	1.94	1.87	2.02	1.94	2.12	1.85	1.94	0.95	7.93	7.80	7.35	7.19	11.73	19.97	5.17
21	0.42	0.42	0.50	0.43	0.63	1.59	1.56	1.89	1.58	1.45	3.61	3.36	10.98	5.02	1.99	11.81	9.49	20.73	12.01	7.58
22	0.32	0.30	0.52	0.43	0.40	1.77	1.76	2.07	1.81	1.85	2.28	1.85	5.93	9.54	3.77	12.23	11.50	35.42	15.93	11.61
23	0.38	0.33	0.92	0.38	0.46	1.76	1.76	2.10	1.91	1.86	1.55	1.46	5.77	6.98	5.01	11.38	12.87	13.92	19.73	18.82
24	0.37	0.32	0.54	0.40	0.48	1.84	1.76	1.79	1.87	1.80	0.93	1.44	4.50	9.93	3.52	9.00	10.35	11.21	22.66	17.86
25	0.37	0.35	0.71	0.36	0.49	2.20	2.16	2.21	2.20	2.05	1.61	1.56	2.88	6.93	5.85	10.92	7.03	20.23	10.22	5.08
26	0.30	0.29	0.56	0.30	0.68	1.55	1.52	2.01	1.62	1.77	2.37	2.17	4.66	5.44	3.82	8.38	6.19	27.25	7.36	6.90
27	0.38	0.38	0.58	0.37	0.50	2.51	2.62	3.55	2.64	3.55	3.53	3.40	12.76	6.61	3.18	26.34	23.15	11.77	13.37	12.56
28	0.38	0.35	0.57	0.38	0.60	2.22	2.27	2.56	2.36	2.69	1.93	1.73	5.69	3.35	4.57	17.57	14.97	33.68	12.50	14.98
Average	0.41	0.46	0.83	0.53	0.62	1.87	1.96	2.83	2.13	2.12	2.45	2.26	4.89	6.58	4.43	11.31	11.31	18.11	14.74	12.02

## Data Availability

The data and codes used in this study are experimental and are available on request from the corresponding author.
